# Two Chemically Stable Cd(II) Polymers as Fluorescent Sensor and Photocatalyst for Aromatic Dyes

**DOI:** 10.3390/polym10030274

**Published:** 2018-03-07

**Authors:** Jun Wang, Jian Wu, Lu Lu, Ai-Qing Ma, Wan-Shan Hu, Wei-Ping Wu, Yu Wu, Yan-Chun Sun, Amita Singh, Abhinav Kumar

**Affiliations:** 1 School of Chemistry and Environmental Engineering, Sichuan University of Science & Engineering, Zigong 643000, China; lulusczg@126.com (L.L.); weipingwuzg@126.com (W.-P.W.); wuyuhlj@163.com (Y.W.); sunyanchun1120@163.com (Y.-C.S.); 2 Guangxi Key Laboratory of Chemistry and Engineering of Forest Products, Guangxi University for Nationalities, College of Chemistry and Chemical Engineering, Nanning 530006, China; wujian2007gx@126.com; 3 Dongguan Key Laboratory of Drug Design and Formulation Technology, Key Laboratory of Research and Development of New Medical Materials of Guangdong Medical University, School of Pharmacy, Guangdong Medical University, Dongguan 523808, China; wanshanhu2018@126.com; 4 Department of Chemistry, Faculty of Science, University of Lucknow, Lucknow 226 007, India; singhamita3010@gmail.com

**Keywords:** chemosensor, photocatalyst, polymer, DOS

## Abstract

Two new 2D Cd(II)-based coordination polymers (CPs), viz. [Cd_2_(H_2_L)_2_(2,2’-bipy)_2_] (**1**) and [Cd(L)_0.5_(phen)·0.5H_2_O] (**2**), have been constructed using ethylene glycol ether bridging tetracarboxylate ligand 5,5′(4,4′-phenylenebis(methyleneoxy)) diisophthalic acid (H_4_L). Both CPs behaved as profound fluorescent sensor for Fe^3+^ ion and nitro-aromatics (NACs), specifically 2,4,6-trinitrophenol (TNP). The stability at elevated temperature and photocatalytic behaviors of both **1** and **2** for photo-decomposition of aromatic dyes have also been explored. An attempt has been made to explore the plausible mechanism related with the decrease in fluorescence intensity of **1** and **2** in presence of NACs using theoretical calculations. Additionally, the probable mechanism of photo catalysis by **1** and **2** to photo-degrade aromatic dyes has been explained using density of states (DOS) calculations.

## 1. Introduction

The existence of traces of hazardous chemicals (HCs) inculcating both organic as well as inorganic compounds after their certain threshold limit in the environment is imposing detrimental effect on environment as well as living creatures [1,2,3,4]. Among organic compounds, the common nitro-aromatic explosives, in particular 2,4,6-trinitrophenol (TNP), can cause a menace due to its explosive nature. It also poses severe health problems to humans and affects the ground water/soil as well [5,6,7,8,9,10]. In addition, in the inorganic cations category, Fe^3+^ is vital for the formation of hemoglobin and muscle and additionally improves brain functions [11]. The excess or deficiency of Fe^3+^ is so detrimental that it may lead to damage to nucleic acids and proteins [11,12,13,14,15]. Thus, a highly effective and fast technique to detect trace amount of Fe^3+^ over other metal ions is of much concern. Nowadays, luminescent coordination polymers (CPs) with open coordination sites have attracted much attention, as these can efficiently interact with various metal ions and hence offer a useful methodology for the development of luminescence sensors and heterogeneous catalysts [16,17,18,19,20]. The incorporation of carboxylate groups is an effective way to construct CPs with open coordination sites [21]. Cohen et al. reported a series of CPs based on 2-phenylpyridine-5,4′-dicarboxylicacid (dcppy) and was the first group who performed cyclometalation post-synthetic modification reactions on these CPs [21].

The aromatic organic compounds deployed as dyes in the past few years have been widely used as chemicals in many industries, but they are hard to biodegrade, thereby causing serious environmental pollution [22]. Thus, the degradation of dye molecules into relatively less hazardous products is very important for environmental protection. Mostly CPs behave like insulators, but some semiconducting CPs are also reported and it has theoretically been proposed that syntheses of numerous CPs displaying semiconducting properties are possible, which can catalyze the photo-degradation of aromatic dyes [23,24,25,26,27,28,29]. Therefore, the proper design and synthesis of porous CPs displaying multifarious molecule-based applications is highly desirable but it is still a huge challenge to chemists [30,31,32,33,34,35].

Keeping these characteristics of CPs in mind and in our incessant pursuit for the designing and syntheses of new CPs that can display dual properties, i.e., as fluorescent sensor for nitro-aromatics/ions and photocatalysts for the photo-decomposition of aromatic dyes [3,4], in the presented work, we used an unexploited 5,5′-(4,4′-phenylenebis(methyleneoxy)) diisophthalic acid (H_4_L) to fabricate Cd(II)-based CPs comprising of 2,2′-bipyridyl and 1,10-phenanthroline as ancillary ligand. The CPs reported in the presented investigation comprises a 2D framework and have been used as a fluorescent sensor for Fe^3+^ ions and 2,4,6-trinitrophenol (TNP). In addition, the photocatalytic properties of these CPs have been explored for the photo-decomposition of aromatic dyes, viz. methyl violet (MV) and Rhodamine B (RhB). The results of these investigations are presented herein.

## 2. Materials and Method

### 2.1. Chemicals and Instrumentation

All chemicals were obtained from commercial sources and used without further purification. All the measurements have been performed on equipment mentioned in our previous report [31].

### 2.2. X-ray Crystallography

The intensity data for single crystalshave been collected on Bruker SMART APEX diffractometer (Bruker, WI, USA) using graphite monochromated MoΚα radiation (λ = 0.71073 Å) by using an ω-scan technique. For both structures, the absorption effect intensities were corrected using SADABS. The structures were solved by direct method (SHLEXS-2014, Sheldrick, G.M, England, UK) and refined using full-matrix least-squares procedure based on *F*^2^ (Shelxl-2014) [36]. All non-hydrogen atoms were refined anisotropically while hydrogen atoms were placed onto calculated positions and refined using a riding model. Crystallographic details and selected bond dimensions for **1** are listed in Tables S2 and S3. CCDC number: 1817769–1817770.

### 2.3. Synthesis of [Cd_2_(H_2_L)_2_(2,2′-bipy)_2_]

A mixture of H_4_L (0.05 mmol, 0.027 g), 2,2′-bipy (0.019 g, 0.1 mmol), Cd(NO_3_)_2_·4H_2_O (0.15 mmol, 0.046 g) and 6 mL of 1:1 (*v/v*) acetonitrile: H_2_O mixture was stirred for 30 min and then transferred and sealed in a 25 mL Teflon-lined reactor and heated to 120 °C for 72 h. Thereafter, the reactor was cooled to room temperature at a rate of 5 °C/h to obtain yellow block crystals of **1** in 71% yield based on cadmium. IR: 3068(m); 1702(m); 1662(vs); 1531(vs); 1434(vs); 1388(m); 1274(m); 1170(m); 1023(v); 817(m); 759(v); 725(m).

### 2.4 Synthesis of [Cd(L)_0.5_(phen)·0.5H_2_O]

The synthesis procedure of **2** was analogous to that of **1**, except that 2,2′-bipy was replaced by phen (0.1 mmol, 0.020 g). Colorless block crystals of **2** were obtained in 64% yield based on cadmium. IR: 3068(m) (Figure S1); 1673(m); 1660(vs); 1542(vs); 1433(vs); 1371(m); 1262(m); 1170(m); 1039(v); 815(m); 756(v); 723(m).

### 2.5. Computational Protocols

The plausible mechanism related with the decline in emission intensity of CPs **1** and **2** in the presence of nitro-aromatics have been proposed with the aid of density functional theory (DFT) calculations. For this the nature of Highest Occupied Molecular Orbital (HOMO) and (Lowest Unoccupied Molecular Orbital) LUMO of different analytes, CPs **1** and **2** have been assessed through geometry optimization which were calculated using the B3LYP exchange-correlation functional [37,38]. The 6-31G** basis set for all the atoms except Cd was used for geometry optimization. For Cd, CEP-121G basis set was employed. All the calculations were performed using Gaussian 09 program (Burant, J.C. et al., Gaussian, Inc., Wallingford, CT, USA) [39]. GaussSum 3.1 (Burant, J.C. et al. Gaussian, Inc.) was used to obtain density of state (DOS) plots [40].

## 3. Results and Discussion

### 3.1. [Cd_2_(H_2_L)_2_(2,2′-bipy)_2_] 

**Figure 1:**
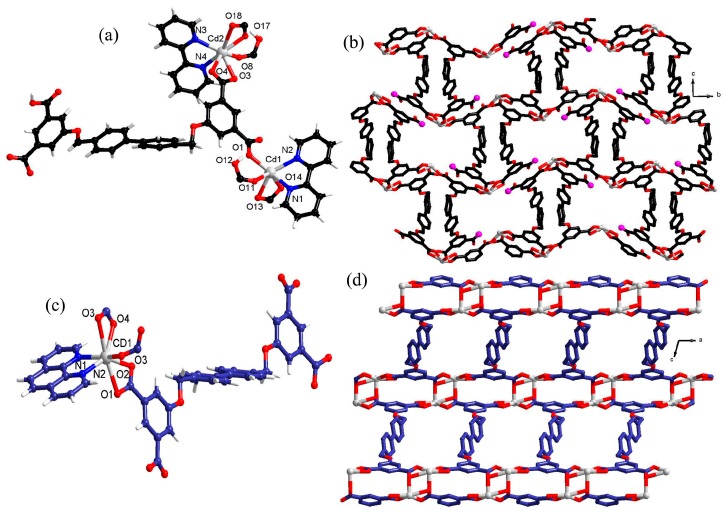
(**a**) The view of local coordination geometry of Cd(II) center and ligand in **1**. (**b**) The 2D layer in **1** as viewed slightly off the *b* axis. The hydrogen atoms have been omitted for clarity; the uncoordinated carboxyl groups pointing to the interior of pores (pink color). (**c**) The local coordination environment around Cd(II) in **2**. The hydrogen atoms have been omitted for clarity. (**d**) The 2D ladder-like layered architecture in **2**.

Single-crystal X-ray diffraction analysis revealed that the CP **1** crystallizes in monoclinic space group *P*2_1_/c and possess a 2D layer structure. The asymmetric unit consists of twoindependent Cd^2+^ ions, two partially deprotonated H_2_L ligands and two bipy ligands (Figure 1a). The Cd1 ion possess a distorted octahedral coordination geometry where each Cd1 ion is coordinated to two N atoms from one 2,2′-bipy ligand and four O atoms from two partially deprotonated H_2_L ligands (Scheme S1a,b). The carboxyl groups of the H_4_L ligand display two types of coordination modes, viz. chelating and monodenate modes. The two Cd1 ions are connected by two carboxylate groups in an alternative chelating and monodenate mode to form a binuclear unit [41]. In the unit, the distance between the two Cd(II) ions is 8.173 Å. Cd2 is hepta-coordinated and the seven-coordinated geometry is satisfied by two N atoms from one 2,2′-bipy ligand and five O atoms from two partially deprotonated H_2_L ligands (Scheme S1a,b). It is noteworthy that H_4_L ligand is partially deprotonated with two carboxylic groups connect to four Cd(II) atoms, which may function as the potential active site (see Figure 1b). Interestingly, there are uncoordinated carboxyl groups pointing to the interior region of pores [10] (Figure 1b). Each L^4−^ ligand links four Cd(II) ions to give rise to a two-dimensional (2D) layer (Figure 1b). When viewed along *b* axis, the two-dimensional layer looks like a wavy chain, which is decorated by the 2,2′-bipy ligands.

### 3.2. [Cd(L)_0.5_(phen)·0.5H_2_O] 

The single crystal X-ray diffraction analysis indicates that complex **2** also comprises a 2Dlayer structure. Within the asymmetric unit, there is one independent Cd^2+^ ion, half L^4−^ ligand, a phen ligand and a half free water molecule. As presented in Figure 1c, the Cd(II) ion is coordinated to two N atoms of phen ligand and five O atoms from one deprotonated H_4_L ligand thereby forming a pentagonal bipyramid coordination geometry around Cd(II). The carboxylate groups of L^4−^ ligand adopt μ_2_-η^1^:η^1^ chelating and μ_2_-η^2^:η^1^ bridging modes, which generated a 2D ladder-like layer (Scheme S1c and Figure 1d). 

### 3.3. Luminescence Sensing

**Figure 2:**
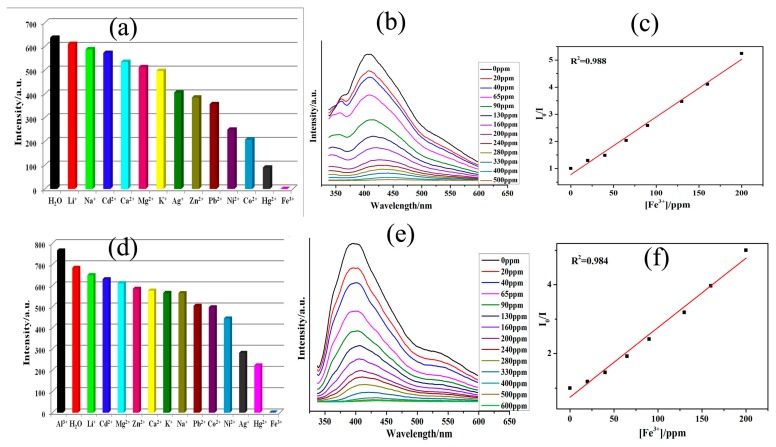
(**a**) The photoluminescence intensity of **1** dispersed in different metal ions solutions (λ_ex_ = 280 nm); (**b**) the emissive response spectra of **1** for aqueous Fe^3+^ solution with different concentrations; (**c**) the Stern–Volmer plot for Fe^3+^ in presence of **1**; (**d**) the Photoluminescence intensity of **2** dispersed in different metal ions solutions (λ_ex_ = 280 nm); (**e**) the emissive response spectra of **2** for aqueous Fe^3+^ solution with different concentrations; and (**f**) the Stern–Volmer plot for Fe^3+^ in presence of **2**.

The structure of 1 was determined by single crystal X-ray diffraction analysis and characterized by IR spectroscopy and thermogravimetric analysis (Figures S1 and S2). The solid-state luminescent properties of CPs **1** and **2** and H_4_L ligandwere examined at room temperature (Figure S3). Since both **1** and **2** are comprisied of d^10^ configuration based Cd^2+^ ions, they both show strong emission bands at 405 nm and 388 nm (λ_ex_ = 290 nm), respectively, which can be ascribed to the π*→π or π*→n transitions corresponding to the H_4_L ligand (λ_e_ = 345 nm; λ_ex_ = 290 nm) [42]. The appropriate luminescent properties are the prerequisite for a CP to behave as luminescent sensors to detect metal ions. Since the detection of metal ions is usually conducted in liquid phase, various solvents are chosen to test their influences on the initial luminescence of the CPs. It was observed that varied samples M^z+^@**1/2** prepared by suspending the CPs**1/2** in aqueous solutions of different metal nitrates (M(NO_3_)_x_) exhibited noticeably diversified photoluminescence properties (Figure S4). Among the different systems, Al^3+^@**2** displayed small enhancement in luminescence (Figure 2d and Figure S5). Notably, the luminescent intensities of both **1** and **2** were selectively quenched in presence of Fe^3+^ ions. Additionally, it was observed that with rise in concentration of Fe^3+^, the emission intensities of both **1** and **2**alleviated (Figure 2b,e). To examine the sensitivity of CPs **1** and **2** towards Fe^3+^ ion, the concentration gradient experiments were executed by varying concentrations of Fe^3+^ solutions in the concentration range from 0 to 500 and/or 600 ppm (Figure 2b,e). These experiments indicated that luminescence intensities of Fe^3+^@**1/2** progressively decreased with rise in concentration of Fe^3+^ [43,44,45,46]. 

Moreover, interesting results have been obtained with CP **1**. The addition of Fe^3+^ ions to **1** results in disappearance of the original emission band (Figure 2b). The disappearance of the band can be explained on the basis that Fe^3+^ ions interact with the –COOH groups of the H_4_L ligand in **1** as well as the intramolecular charge-transfer phenomenon [46]. 

The Stern–Volmer plots for Fe^3+^ are almost linear at low concentrations with the *K*_sv_ value of 8.59 × 10^3^ M^−1^ (Figure 2c). The *K*_sv_ value iscommensurate to some earlier reported MOF-based sensors, such as [La(TPT)(DMSO)_2_]·H_2_O (1.36 × 10^4^ M^−1^) [44], [La(TAIP)(DMF)_2_](DMF)_0.5_ (8.86 × 10^3^ M^−1^) [45], and Eu-MOF-LIC-1 (2.87 × 10^4^ M^−1^) [46]. The Fe^3+^ ion detection limit has been calculated as 0.75 and 0.79 ppm for **1** and **2**, respectively.

**Figure 3:**
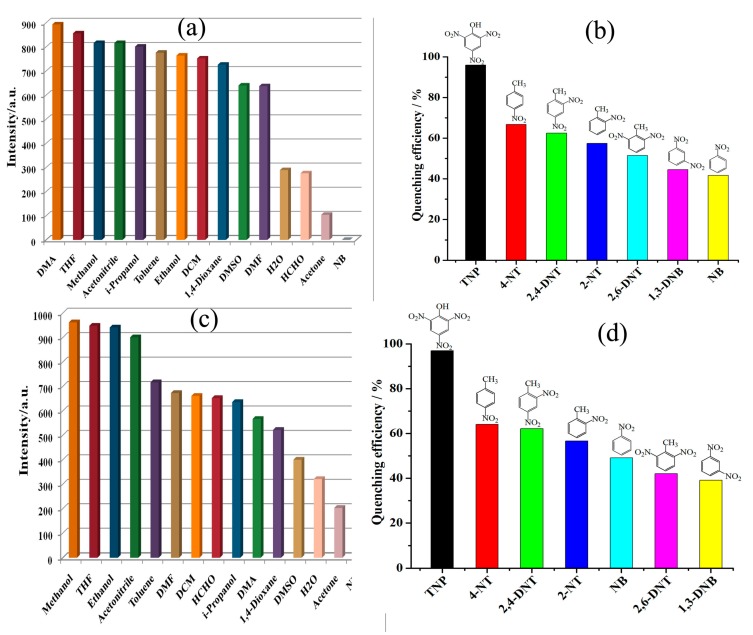
(**a**,**c**) The emission intensities of CPs **1** and **2** dispersed in different solvents (λ_ex_ = 290 nm); and (**b**,**d**) emissive response spectra of **1** and **2** for TNP in DMF solutions, respectively.

The fluorescence responses of **1** and **2** towards small molecules were also measured in *N,N*-Dimethylformamide (DMF) suspensions of **1** and **2** [47,48,49]. The experiments indicated that, among different solvent molecules, nitrobenzene (NB) was having good capacity to decrease the photoluminescent emissions of **1** and **2** (Figure 3a–c and Figures S6 and S7). Thus, the DMF suspension of both **1** and **2** were selected to sense variety of nitro-aromatic compounds (NACs), viz., 2,4,6-trinitrophenol (TNP), 2,4-dinitrotoluene (2,4-DNT), 2,6-dinitrotoluene (2,6-DNT), 2-nitrotoluene(2-NT), 4-nitrotoluene (4-NT), and 1,3-dinitrobenzene (1,3-DNB). The experiments indicated that the increased incorporation of TNP in step-wise manner to the dispersions of **1** and **2** led to appreciable decrement in fluorescence intensities (Figure 3b,d) [50,51,52,53,54,55]. However, in comparison to TNP, other NACs, viz., NB, 1,3-DNB, 2,4-DNT, 2,6-DNT, 2-NT and 4-NT, displayedrelatively small quenching effect (Figures S8–S31). The fluorescence quenching efficiencies of both **1** and **2** were further analyzed using the Stern–Volmer (S–V) equation, (*I*_0_/*I*) = *K*_sv_[Q] + 1 [22]. From the linear fitting of the S–V plots (Figures S32 and S33), the calculated *K*_sv_ value for TNP was found to be 2.85 × 10^3^ M^−1^ for **1** and 2.25 × 10^3^ M^−1^ for **2** (Table S1). These *K*_sv_ values are almost parallel to those of the previously reported MOF based sensors, such as UIO-67-dcppy (2.9 × 10^4^ M^−1^) [51], [Cd(NDC)0.5(PCA)] (3.5 × 10^4^ M^−1^) [52], [Tb(1,3,5-BTC)] (3.42 × 10^4^ M^−1^) [53] and Zn-TCPP (3.59 × 10^4^ M^−1^) [54]. Based on 3δ/slope, the TNP detection limits were 0.86 and 0.94 ppm for **1** and **2**, respectively [56,57,58,59]. Hence, the results show that both **1** and **2** can be utilized to detect nitroaromatics with different electron-withdrawing –NO_2_ group. 

In addition to experiments, the plausible mechanism associated with the alleviation in fluorescence intensities of **1** and **2** in presence of different NACs have been addressed with the help of theoretical calculations. The HOMO–LUMO energies of the NACs along with CPs **1** and **2** were calculated using density functional theory (DFT) at the B3LYP level (Table 1 and Figure S34a,b). The possible reason behind quenching may be the electron transfer operating from the framework of **1** or **2** to the LUMO of the analytes [5,6,7,8,22]. The phenomenon of electron transfer will happen only when the LUMO of the donor MOF **1** or **2** will have higher energy in comparison to LUMO of the acceptor analytes. The LUMO energies of H_4_L, **1**, **2** and NACs presented in Table 1 indicate that the LUMOs of all NACs are at comparatively lower energy scale in comparison to **1** and **2**, which facilitates the electron transfer from **1** or **2** to NACs. However, the observed order of quenching in the emission of **1** or **2** by these NACs is not in full agreement with the corresponding LUMO energies of NACs, which indicates that the electron transfer phenomenon is not the sole mechanism for the quenching in intensity. Hence, alongwith the electron and energy transfer processes, there may be the possibility of weak interaction operating between CPs and NACs which may also be playing rolein the decrease in the emission intensities of both the CPs [60,61,62,63,64,65,66,67,68,69,70,71]. Additionally, there is also the possibility that there are certain constraints related to transition probability of both the CPs, for instance the NACs inhibits linker motions (at the excited state) in CPs, which might be responsible for the decrement in emission intensity [68,69,70,71].

The UV/Vis absorption spectra for Fe(NO_3_)_3_ and TNP solutions have been recorded (Figure S35). The electronic absorption spectra of Fe^3+^ and TNP solution display large overlap with the excitation spectrum of H_4_L. Therefore, the competition absorption of excitation wavelength (290 nm) energy between Fe^3+^ aqueous solution and both the CPs may be responsible for the quenching effect.

### 3.4. Diffuse-Reflectance UV/Vis Spectroscopy

To obtain diffuse-reflectance spectra, the UV/Vis spectra for **1** and **2** were recorded in solid state at RT (room temperature). For both **1** and **2**, the spectra comprise bands in the UV region (Figure S36). The intense absorption band at ~300 nm may be arising because of the π‒π* transitions of the ligand. In the diffuse reflectance spectroscopy (DRS), the scattered radiation is collected by excluding specularly reflected light which matches closely with the Kubelka–Munk function *F*(*R*)=(1−*R*)^2^/2*R* [10d]. The energy band gaps (*E*_g_) which had been calculated by extrapolating the linear region of absorption edge comes equal to 2.83 and 3.03 eV for CPs **1** and **2**, respectively (Figure S37). These band gap parameters indicate the semiconducting nature of both the CPs (Figure S37). The band gaps of both **1** and **2** indicate that both may display absorption responses towards UV radiation and concomitantly can have potential to behave as photocatalysts in photodegradation of organic dyes [72,73,74,75].

### 3.5. Photocatalysis 

**Figure 4:**
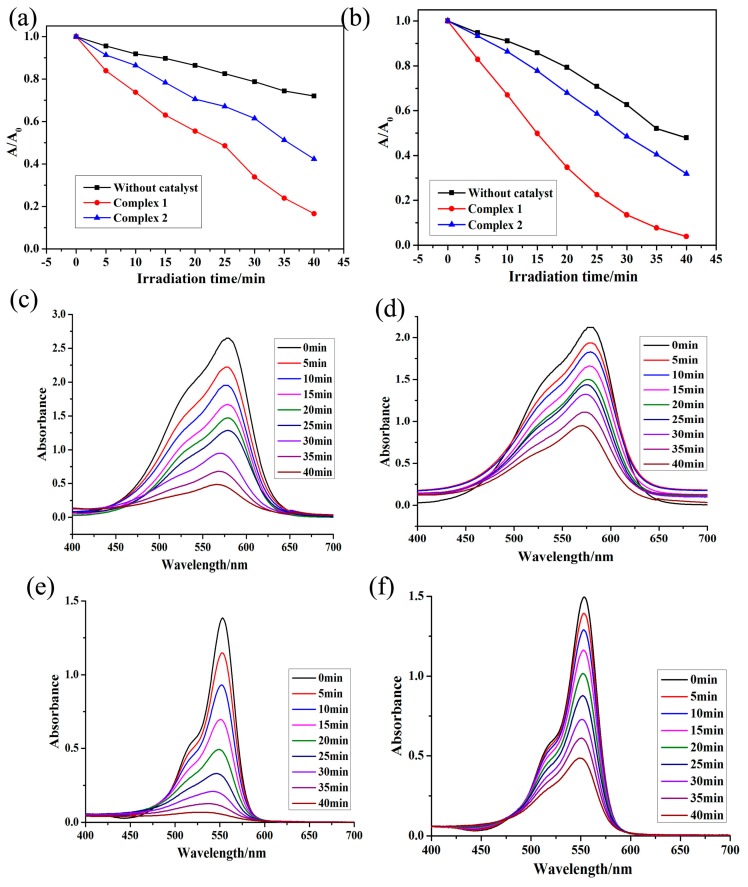
(**a**,**b**) The plot displaying photocatalytic degradation kinetics of MV and RhB in presence ofof CPs **1** and **2**; and (**c**–**f**) UV–vis absorption spectra of the MV and RhB solution during the decomposition reaction under 250 W Hg lamp irradiation in the presence of CPs **1** and **2**, respectively.

The photocatalytic activities of both **1** and **2** were checked by photo-decomposition of the dyes methyl violet (MV) and rhodamine B (RhB) in aqueous medium under UV irradiation using a 250 W Hg lamp. The degradation rates of both the dyes in aqueous medium were checked by observing the change in absorbance of the characteristic absorption bands of both the dyes with time (Figure 4). The non-appearance of any new absorption band in the UV/Vis spectra of dyes indicated their complete decomposition in aqueous medium. The experimental results indicated that the conversion rates of MV and RhB are 83.38% and 96.15%, respectively, in the presence of CP **1**. To judge whether dyes are degrading under UV irradiation even in absence of CPs, the catalytic degradation efficiency of the control experiment (in the absence of CP **1**) was carried out for 100 min (Figures S38 and S39). During this period, in the absence of **1**, the degradation percentages of MV and RhB were 28.02% and 52.07%, respectively. These results validate that the presence of **1** is crucial to photo-degrade MV and RhB. In addition, the photo-decomposition of RhB was more in comparison to MV in presence of **1** under similar reaction conditions. The mechanism related to the photo-degradation of MV and RhB can be substantiated by the fact that UV irradiation of CPs **½** induces excited photo-electron to moves from thevalence band (VB) to the conduction band (CB). The electron deficient holes generated in VB of CPs at Cd(II) sites generates hydroxyl radicals alongwith other oxidants which decomposes the organic dyes [76,77,78,79,80,81,82,83]. After photocatalysis, CPs **1** and **2** were filtered off and observed under an optical microscope. The unchanged Powder X-ray Powder Diffracter (PXRD) patterns for both **1** and **2** indicates that both the CPs remains stable after photo-degrading MV/Rh B. In addition, the diffractogram identity for the sample before and after the photocatalytic experiment would not exclude a partial dissolution or decomposition of the material [78] (Figure S40).

Although the band gap of **2** is adjacent to that of **1**, their photocatalytic performances are distinctively different, which could be influenced by the differences in the CP frameworks [29,30], because of the small difference in the optical band gap between each group of CP (Δ*E*_g_ ≤ 0.12 eV). Its degradation rate is the smallest, only 15.1% for RhB in **2**. Herein, we tentatively surmise that it is possibly caused by the special structure of **2**. As described above, complex **2** is 2D ladder like layer, the dense structure of which will be not conducive to the adsorption and desorption of O_2_/hydroxyl (OH^–^) on its surface and the transport of excited holes/electrons to its surface, to retard the formation of the hydroxyl radicals (**·**OH) and further impede the occurrence of the catalytic to retard the formation of the hydroxyl radicals (**·**OH) and further impede the occurrence of the catalytic reaction. 

**Figure 5:**
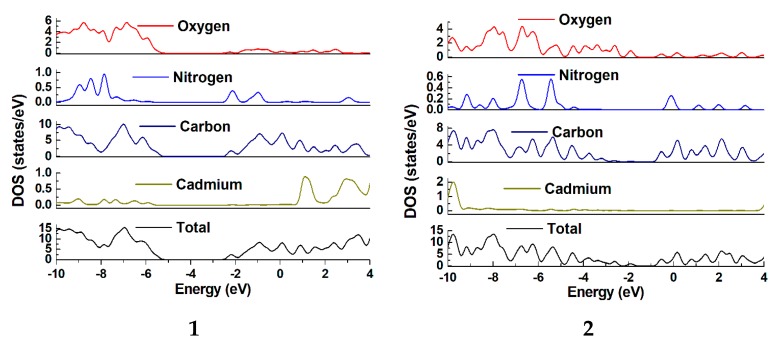
The DOS and partial DOS plots for CPs **1** and **2**.

The probable photo-decomposition mechanism of organic dyes in presence of **1** and **2** have been addressed by band structure calculations using density functional theory method. As evident in Figure 5, the valence band which is lying just beneath the Fermi level in **1** is having contributions from aromatic carbon centers and oxygen centers with small contribution by the Cd(II) and nitrogen centers. Likewise, in **2**, the main contribution for the valence band coming from aromatic carbon centers, nitrogen and oxygen centers with negligible contribution by the Cd(II) centers. In both **1** and **2**, the conduction band lying just above the Fermi level in the range of −2.50 to −1.86 eV is derived from aromatic carbons, nitrogen and oxygen. Therefore, the electronic transition in **1** mainly takes place from the Cd(II) center, nitrogen, oxygen and aromatic region, but in **2** this electronic transition is operating from nitrogen, oxygen and aromatic region while the contribution from Cd(II) is negligible. This non-involvement of Cd(II) center in electronic transition may be the possible reason for the relatively poor photocatalytic property of **2** in comparison to **1**. In a typical photocatalytic process, the samples can be excited to produce electron–hole pairs under visible light irradiation and as band structure calculations reveal that hole moves to metal centers and the electron migrates to aromatic entity. The generation of holes on the d^10^-centers will correspond to its oxidation which is can now oxidize the dye to reduce back to d^10^ configuration again [22].

## 4. Conclusions

In conclusion, the fluorescent CPs **1** and **2** reported herein offered selective sensing property against nitro-aromatics, especially against TNP. Additionally, they can also be used as a photocatalyst for photo-decomposition of aromatic dyes. The quantum chemical calculations prove the existence of both electron and energy transfer processes, in addition to electrostatic interaction between the CPs **1** and **2** and nitro-aromatics, which may be responsible for the unprecedented selective fluorescence quenching. In addition, the density of states calculations revealed that inferior catalytic properties in **2** may arise because of the poor involvement of Cd(II) center in photoexcitation. Hence, the presented investigation proves that not only the choice of polycarboxylates ligand but also the selection of appropriate polypyridyl moiety in the fabrication of CPs plays a crucial role in the development of 2D framework. In addition, by suitable selection of both polycarboxylate linker as well as polypyridyl spacer, one can develop appropriate CPs that can behave as potential sensors and photocatalyst. The presented work will induce stimulus to develop similar CPs for the selective and sensitive detection of nitro-aromatics as well as to synthesize new photocatalysts for organic dye degradation.

## Figures and Tables

**Table 1:** The HOMO–LUMO energies (in eV) for different analytes and 1.

Ligand/Analyte	HOMO	LUMO
1	−5.49	–2.23
2	–1.89	–1.55
2-nitrotoluene (2-NT)	–7.28	–2.32
4-nitrotoluene (4-NT)	–7.36	–2.32
Nitrobenzene (NB)	–7.60	–2.43
2,6-dinitrotoluene (2,6-DNT)	–7.91	–2.87
2,4-dinitrotoluene (2,4-DNT)	–8.11	–2.98
1,3-dinitrobenzene (1,3-DNB)	–8.42	–3.14
2,4,6-trinitrophenol (TNP)	–8.54	–3.55

## Supplementary Materials

The following are available online at http://www.mdpi.com/2073-4360/10/3/274/s1.
