# Supplementary material for: Two Chemically Stable Cd(II) Polymers as Fluorescent Sensor and Photocatalyst for Aromatic Dyes
Source: Polymers (Basel). 2018 Mar 7;10(3):274. doi: 10.3390/polym10030274 (PMC6415190; doi:10.3390/polym10030274)
Supplement: Supplementary file 1 [file polymers-10-00274-s001.docx]

Two Chemically Stable Cd(II) Polymers as Fluorescent Sensor and Photocatalyst for Aromatic Dyes

***Photoluminescence measurements***

The photoluminescence sensing experiments were performed as follows: the photoluminescence properties of **1/2** were investigated in DMF/H_2_O emulsions at room temperature using a RF-5301PC spectrofluorophotometer. The suspensions were prepared by adding 5 mg of **1/2** powders into 3 mL of DMF/H_2_O and then ultrasonic agitation the mixture for 30 min before testing. For the titration experiments of TNP, **1/2** powder (3 mg) was immersed in DMF solutions with the dropped addition of different concentrations of TNP in DMF. The photomultiplier tube (PMT) voltage was 700 V, the scan speed was 1200 nm min^-1^, the slit width of excitation and emission is 5 nm.

***Photocatalytic Method***

The photocatalytic reactions were performed as follows: 30mg of **1/2** were dispersed in 50 mL aqueous solution of RhB/MV (10 mg/L) under stirring in the dark for 30 min to ensure the establishment of an adsorption-desorption equilibrium. Then the mixed solution was exposed to UV irradiation from an Hg lamp (250 W) and kept under continuous stirring during irradiation for 100 min. Samples of 5mL were taken out every 10 min and collected by centrifugation for analysis by UV-Vis spectrometer. By contrast, the simple control experiment was also performed under the same condition without adding any catalysts. The photocatalytic activity studies were carried out in a Shimadzu UV-Vis 2501PC recording spectrophotometer.





Scheme S1 the different coordination modes of H_4_L ligand in this work.

***IR***

In the IR spectra of all the complexes strong absorption bands between 1386 and 1610 cm^-1^ are displayed, which are characteristic peaks for coordinated carboxylate groups.

The existence absorption bands between 1702 cm^-1^ in **1** indicated the undeprotonated H_2_L ligands was observed. that Additionally, there are absorption bands at about 3400 cm^-1^, which can be attributed to the stretching vibrations of the hydroxyl groups in coordinated water molecules in **2** and hydroxyl group in **1** (Fig. S1).


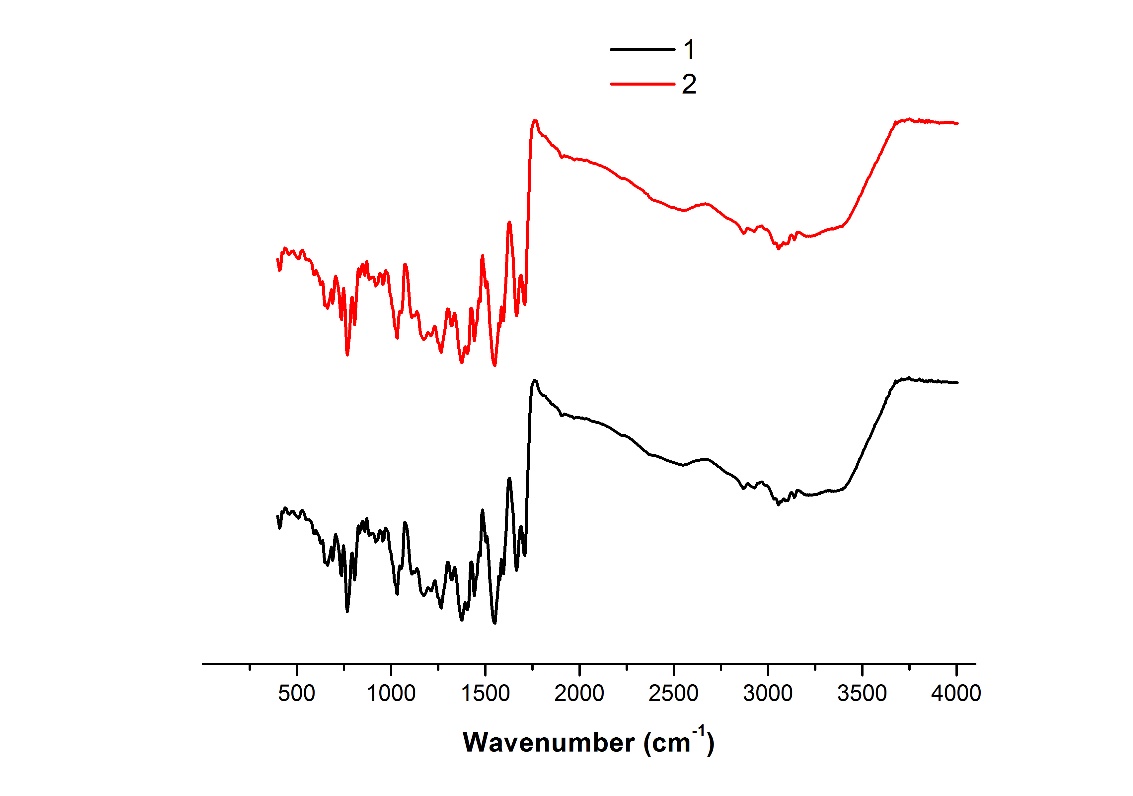


Fig. S1 view of the IR spectra in **1-2**.

***TGA***

Under an atmosphere of nitrogen, the thermal stabilities of the two complexes have been investigated. They exhibit the similar thermostability curves in the temperature range 20 to 800 °C. For **1**, remarkably, it keeps its stability up to 300 °C, after which it begins to decompose. While in 2, it displays a weight loss of 1.4% in the range 80–130 °C, which is ascribed to the departure of free water molecule (obsed: 1.29%).


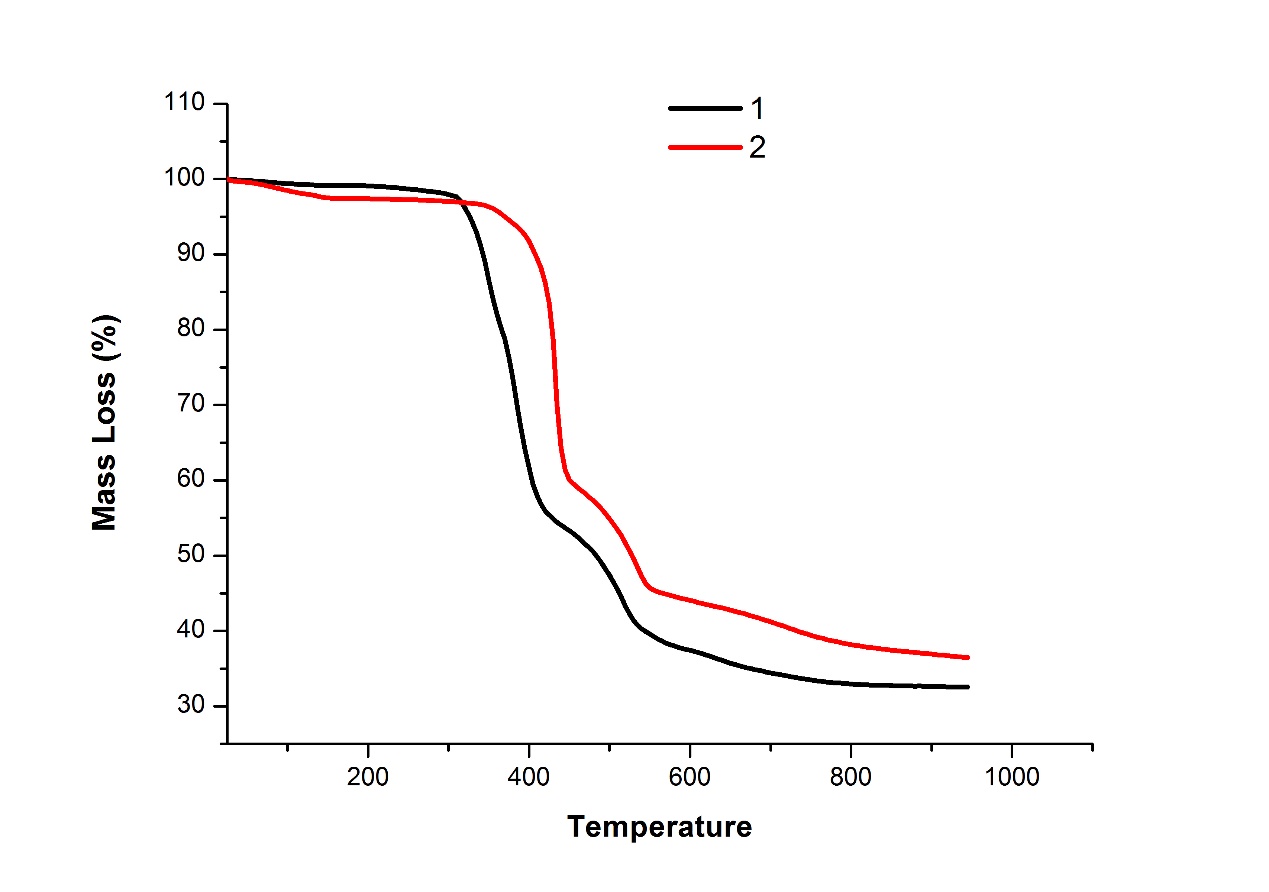


Fig. S2 view of the TGA.
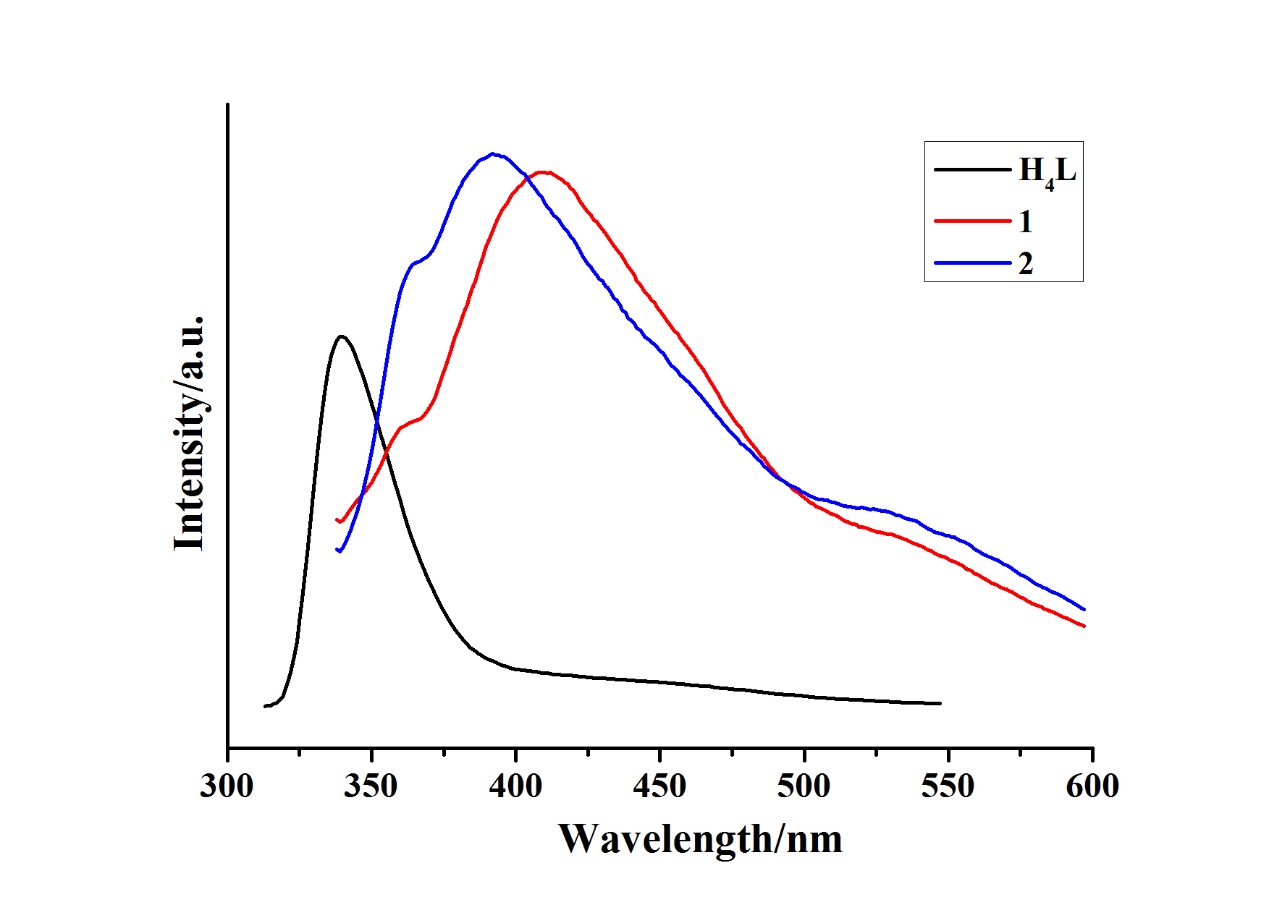


Fig. S3 The photoluminescence spectra of solid samples of H_4_L ligand and **1/2** recorded at room temperature (λ_ex_ =280 nm).


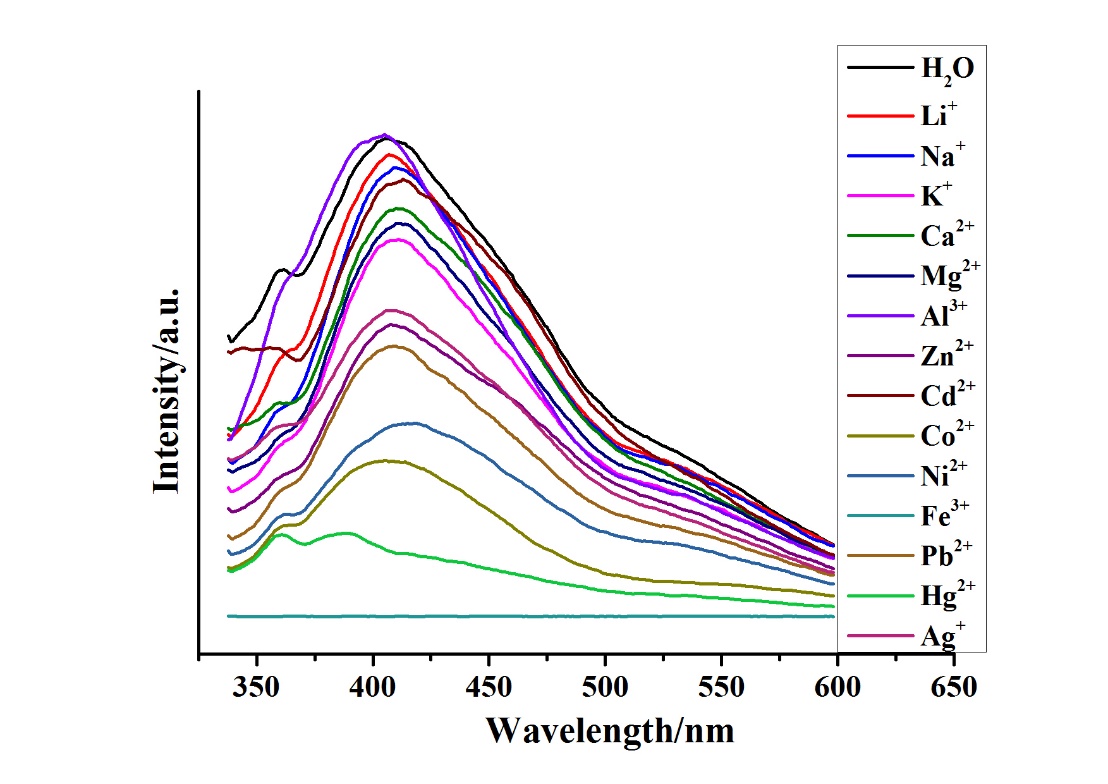


Fig. S4 The photoluminescence intensities spectra of **1** that was dispersed in the solutions of different metal ions.


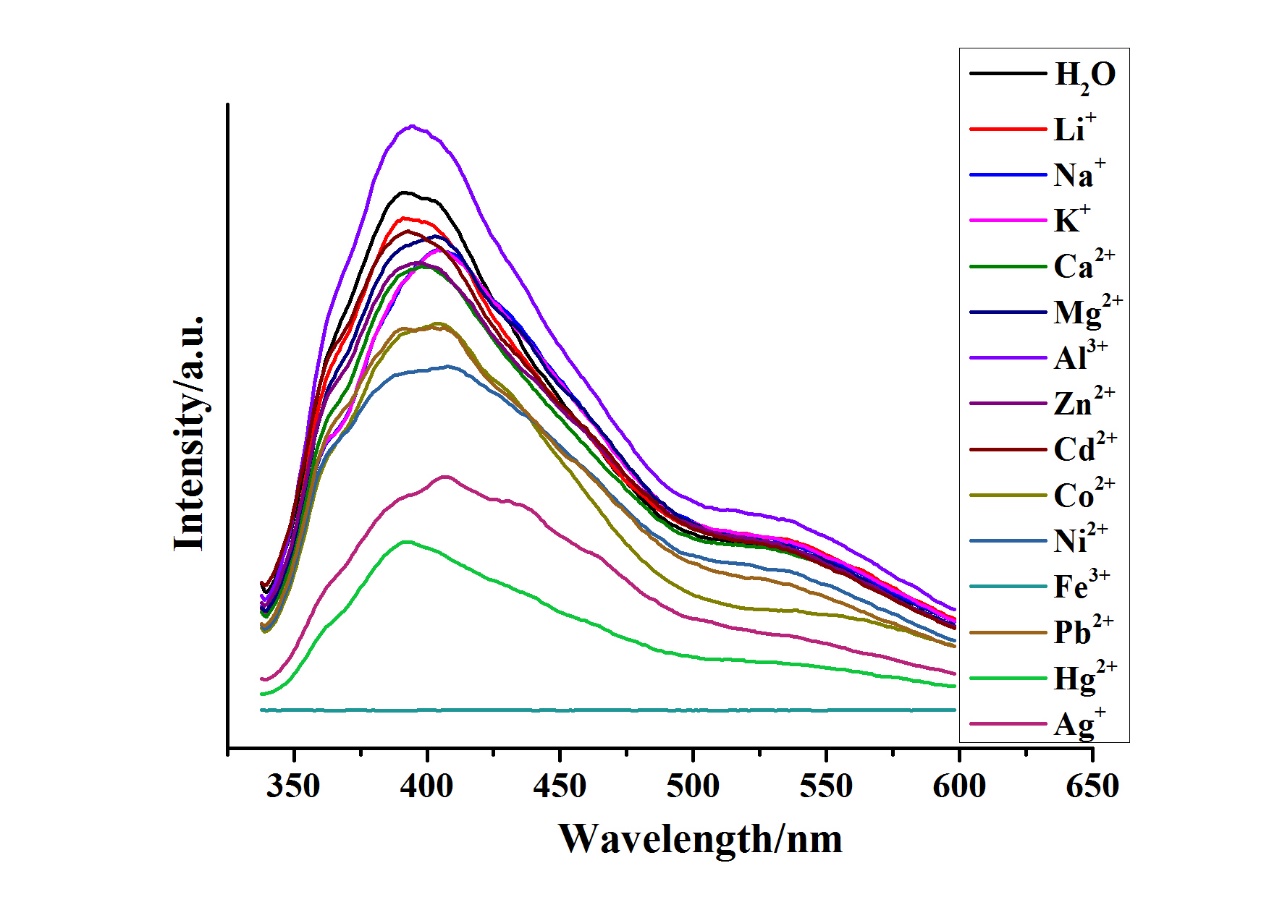


Fig. S5 The photoluminescence intensities spectra of **2** that was dispersed in the solutions of different metal ions.


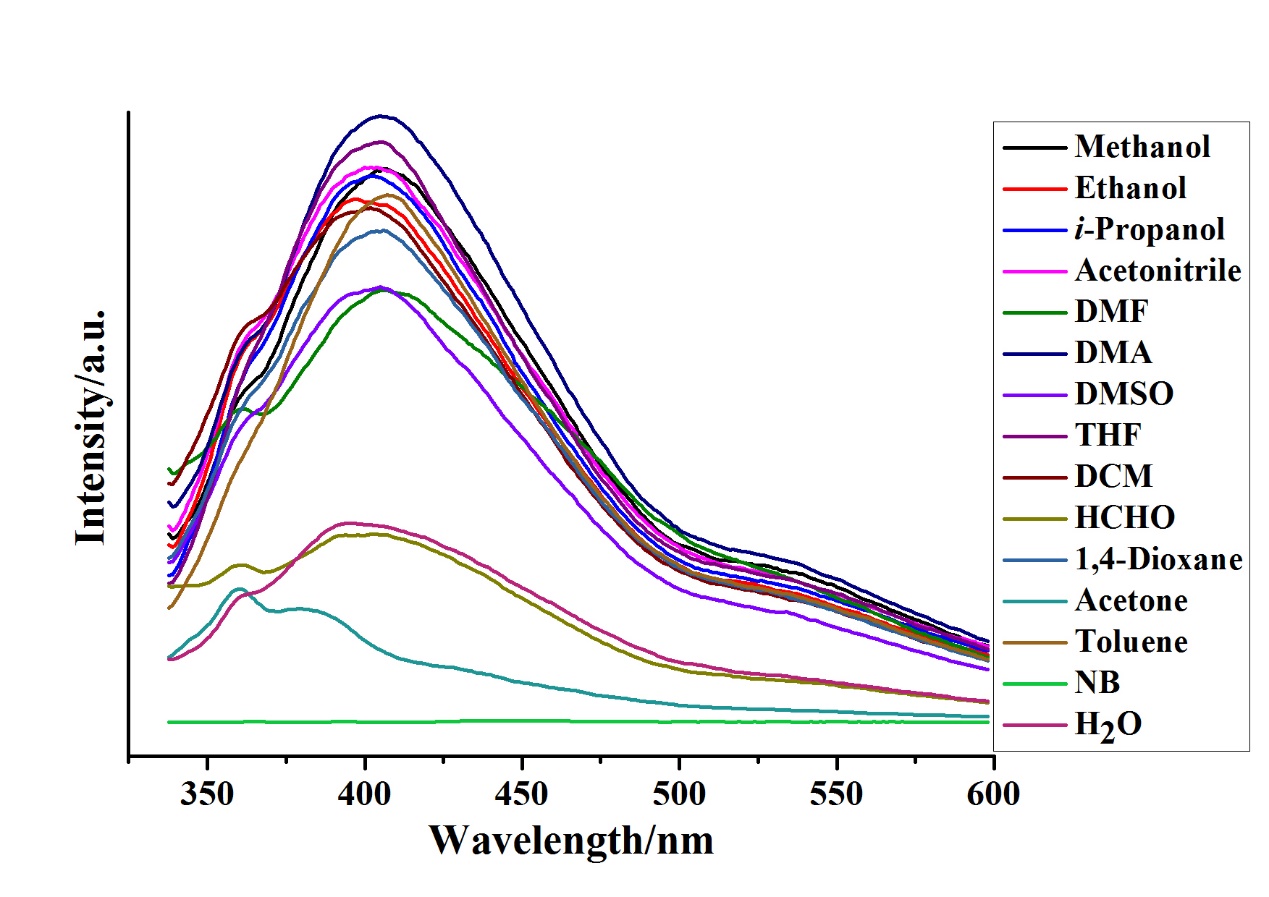


Fig. S6 The photoluminescence intensities spectra of **1** that was dispersed in different solvents.


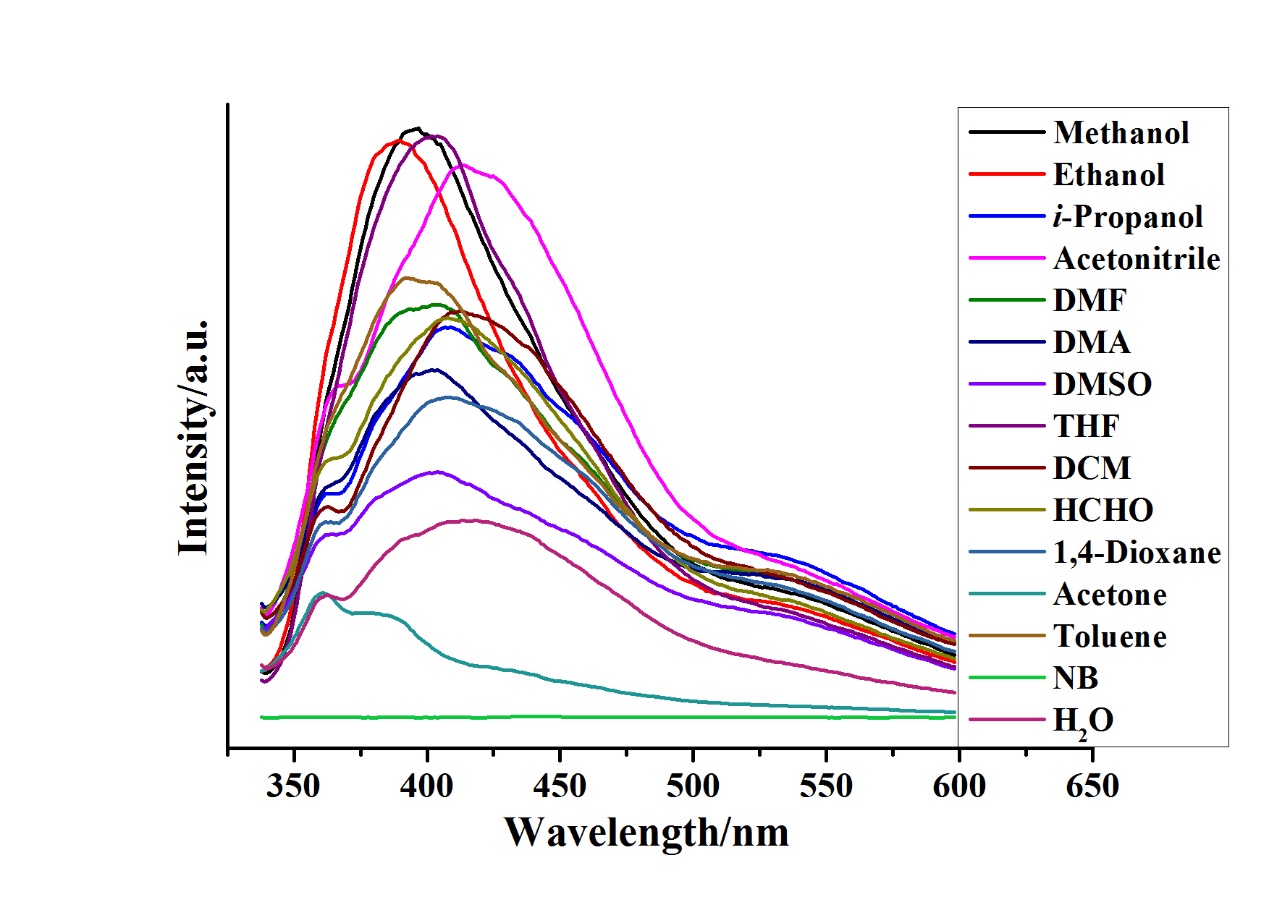


Fig. S7 The photoluminescence intensities spectra of **2** that was dispersed in different solvents.


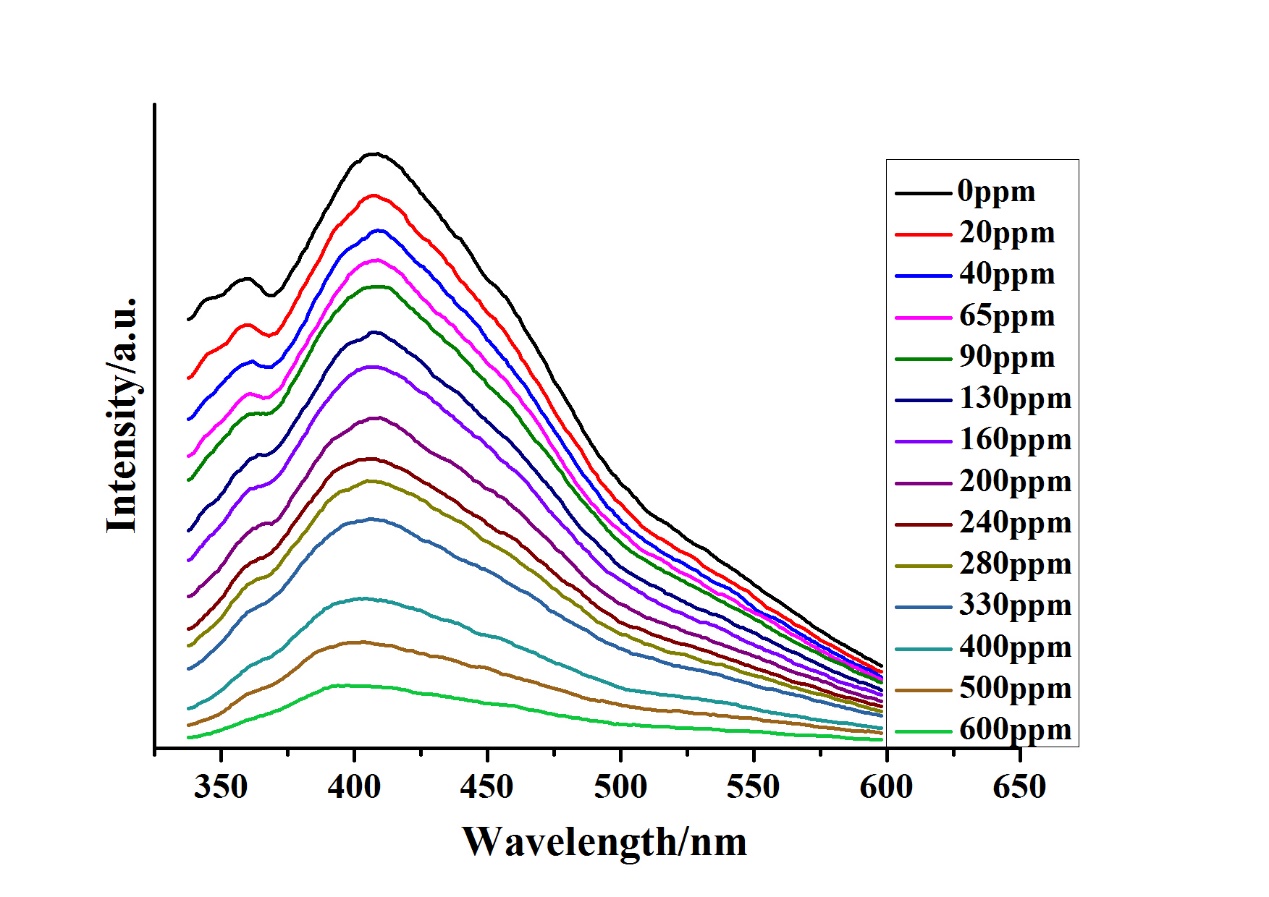


Fig. S8 Luminescent quenching of **1** dispersed in ethanol by the gradual addition of 1 mM solution of 1,3-DNB in DMF.


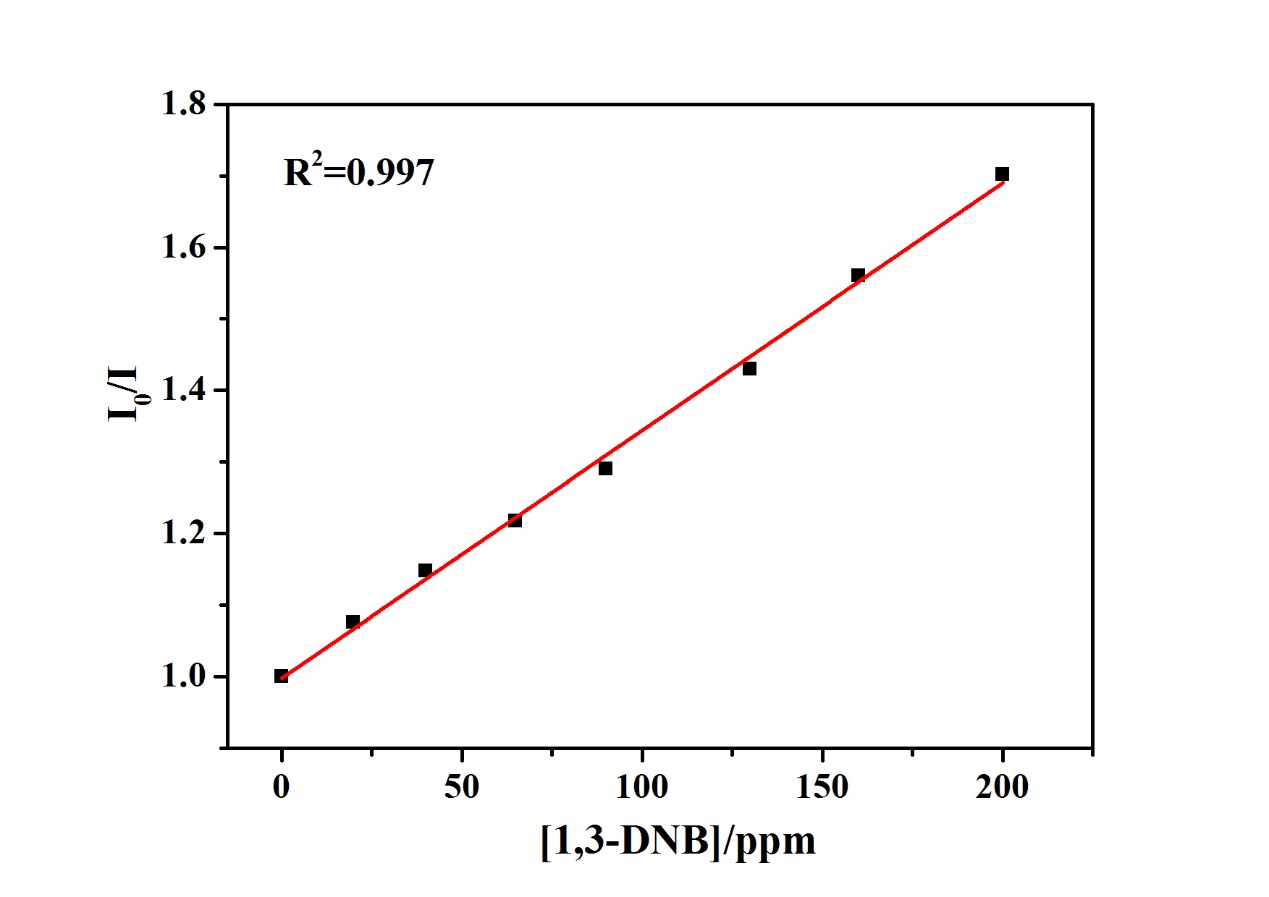


Fig. S9 The Stern–Volmer plot of **1** against 1,3-DNB.


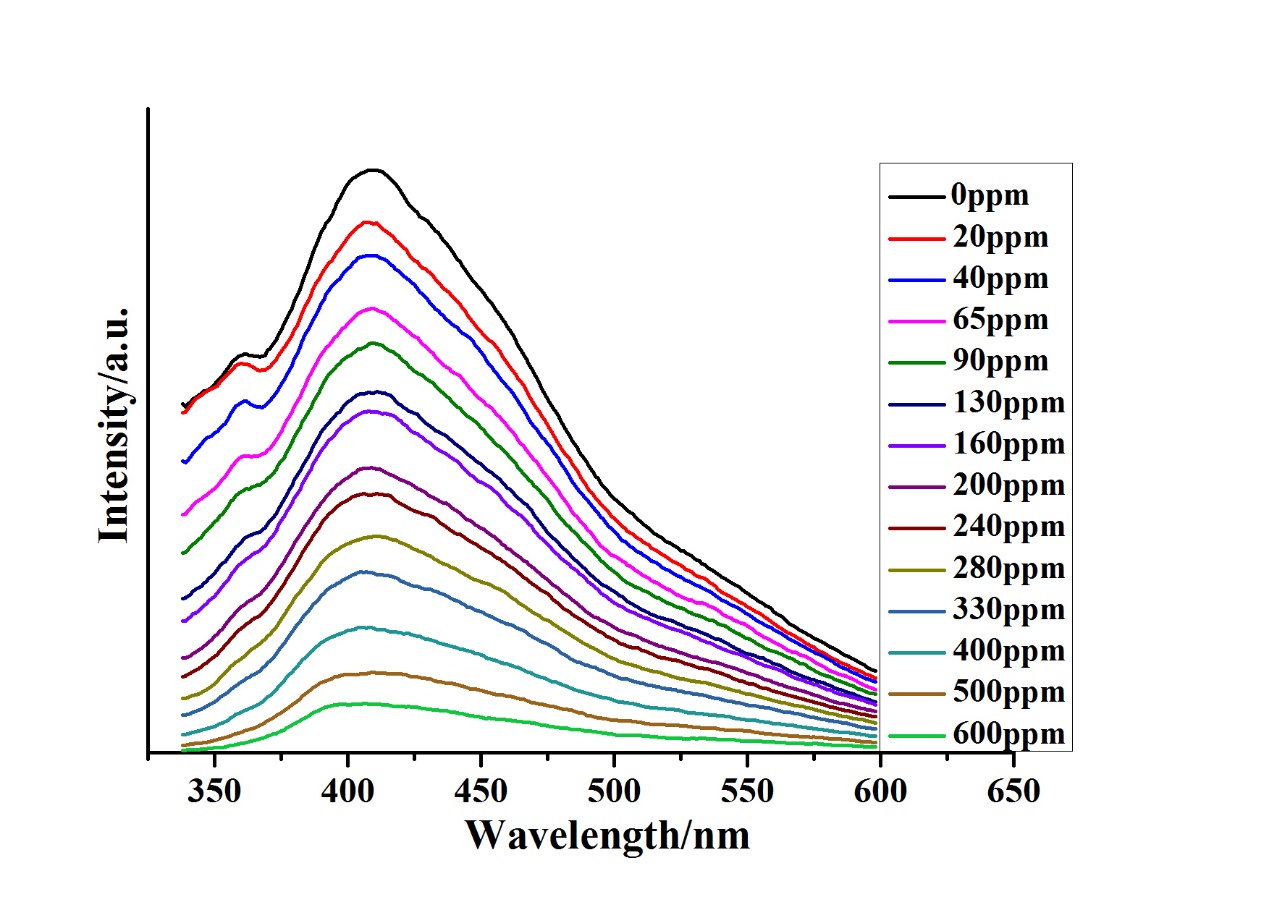


Fig. S10 Luminescent quenching of **1** dispersed in ethanol by the gradual addition of 1 mM solution of 2,4-DNT in DMF.


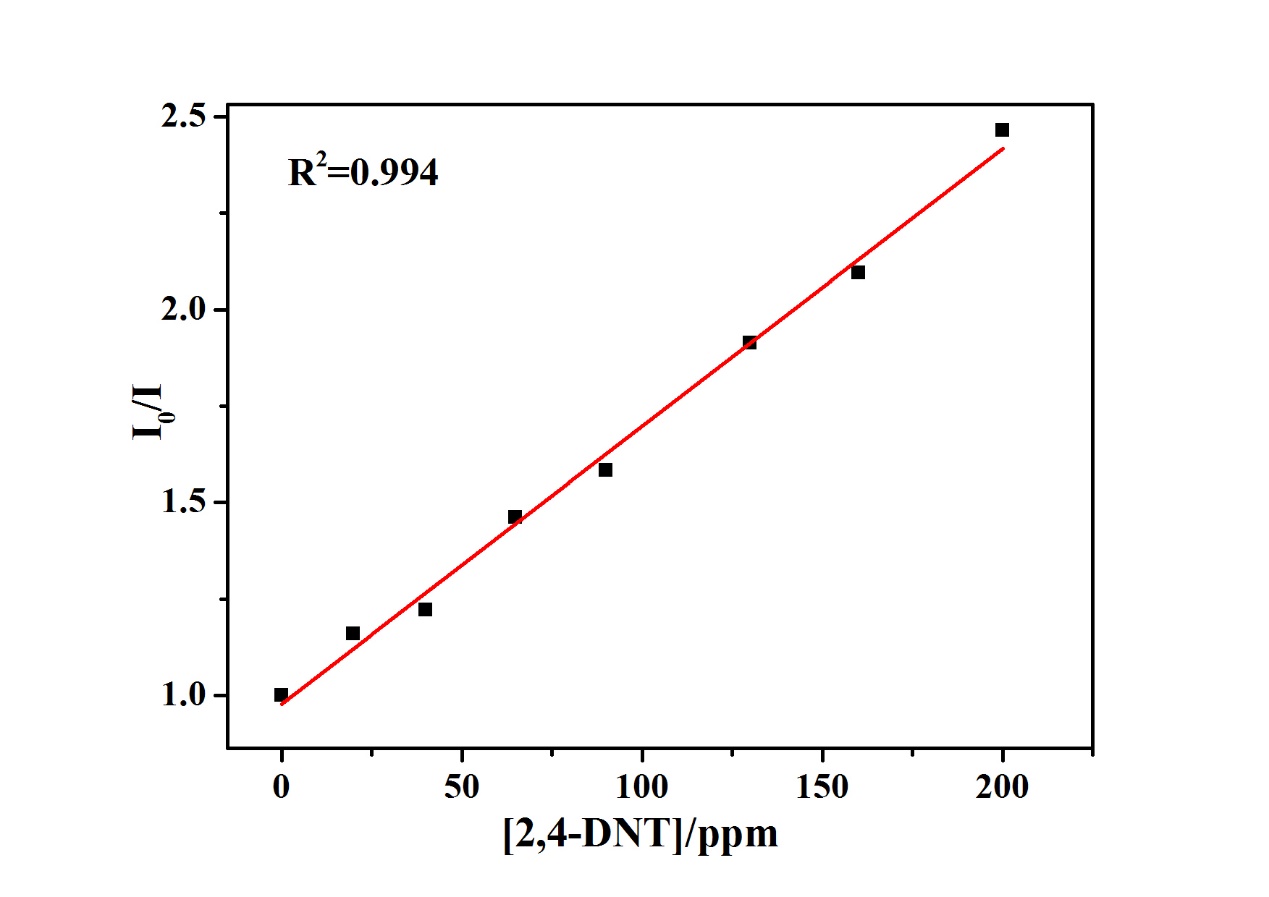


Fig. S11 Stern–Volmer plot for the fluorescence quenching of **1** upon the addition of 2,4-DNT.


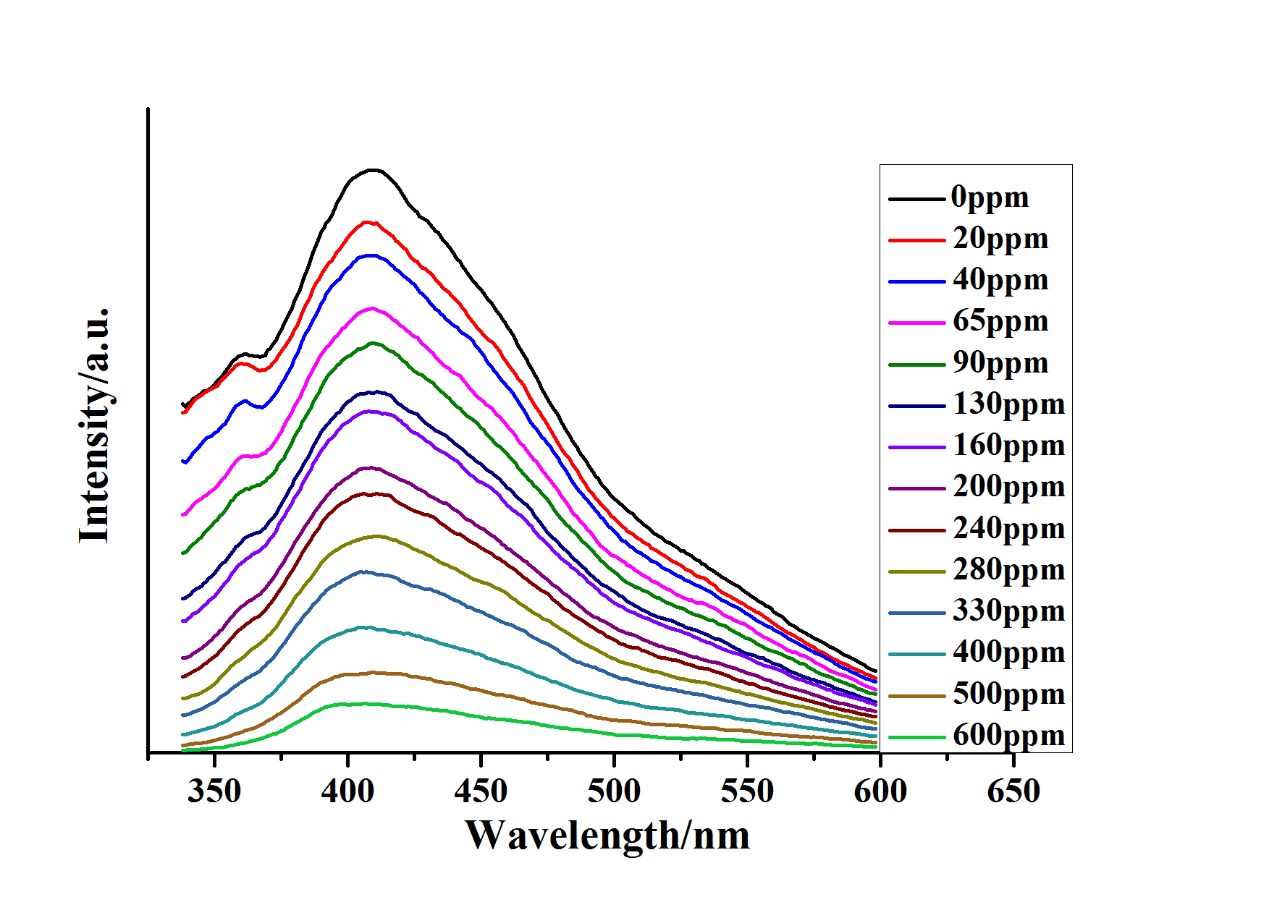


Fig. S12 Luminescent quenching of **1** dispersed in ethanol by the gradual addition of 1 mM solution of 2,6-DNT in DMF.


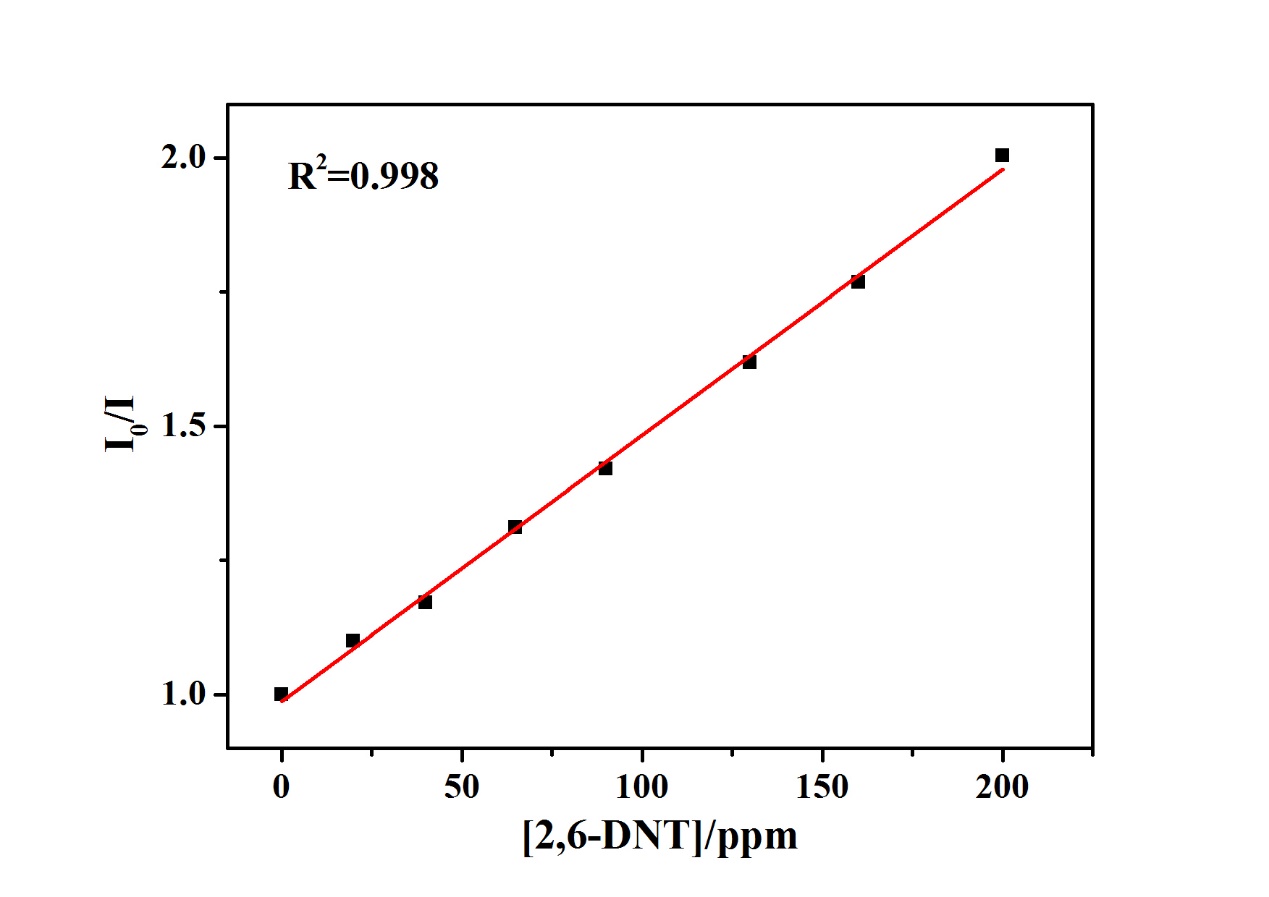


Fig. S13 Stern–Volmer plot for the fluorescence quenching of **1** upon the addition of 2,6-DNT.


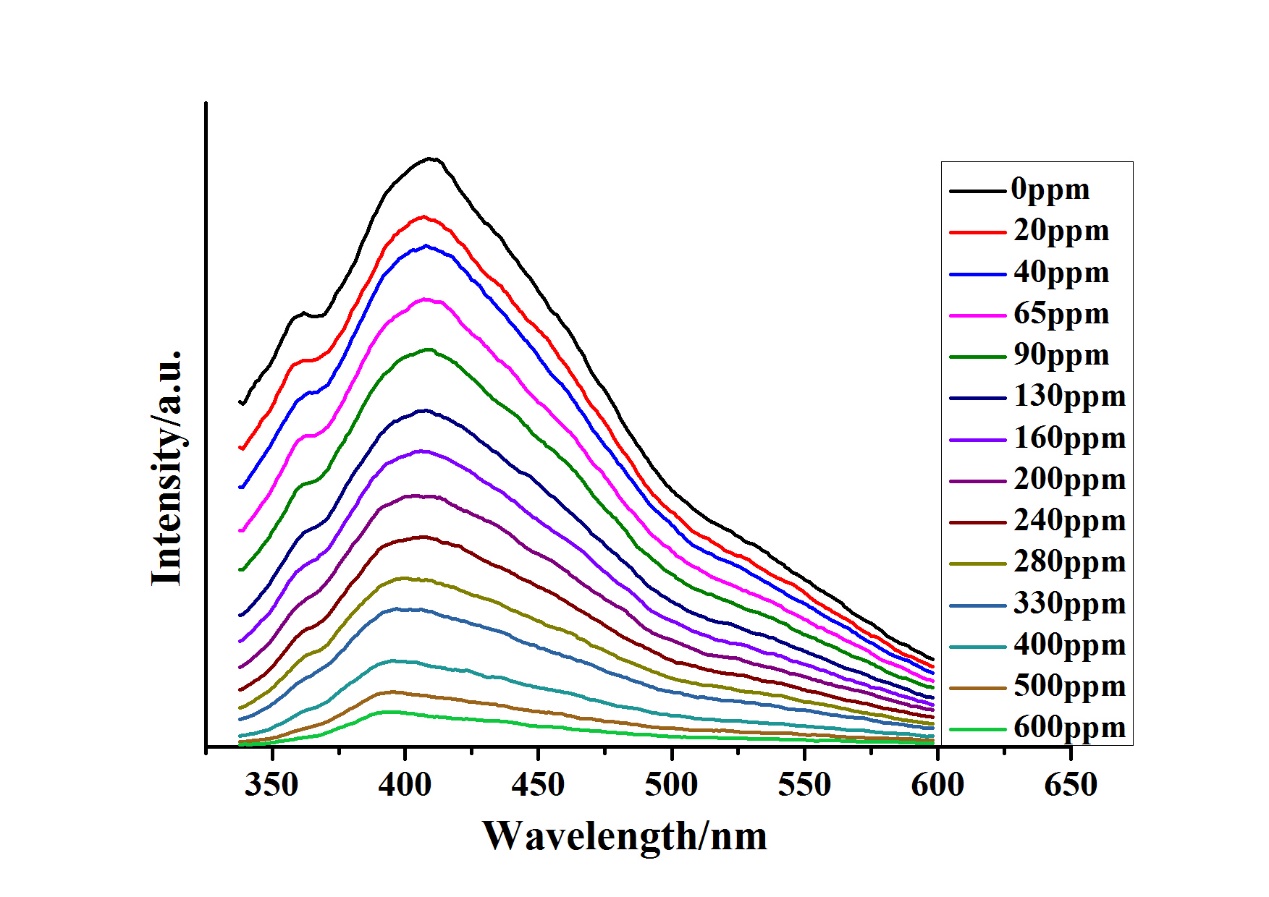


Fig. S14 Luminescent quenching of **1** dispersed in ethanol by the gradual addition of 1 mM solution of 2-NT in DMF.


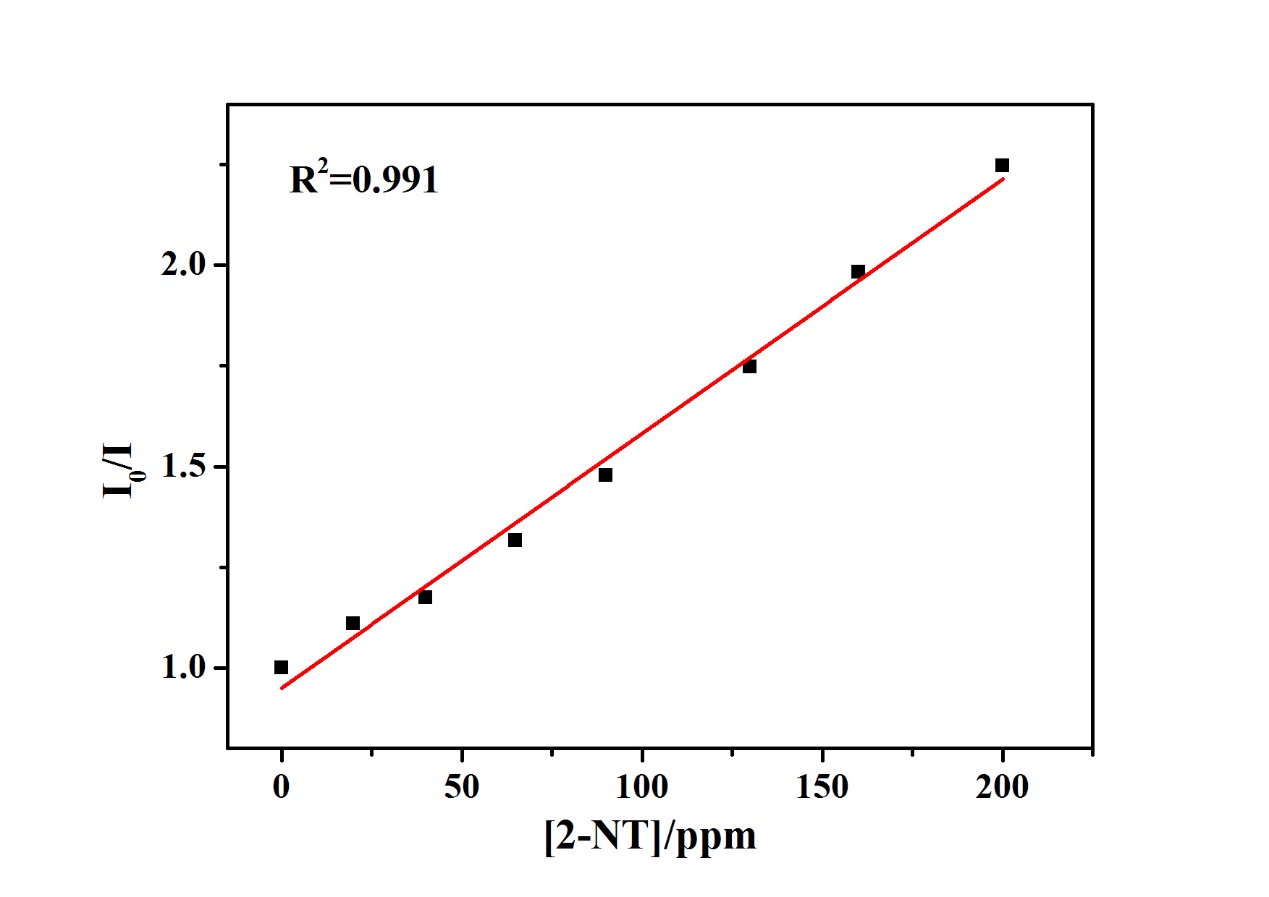


Fig. S15 Stern–Volmer plot for the fluorescence quenching of **1** upon the addition of 2-NT.


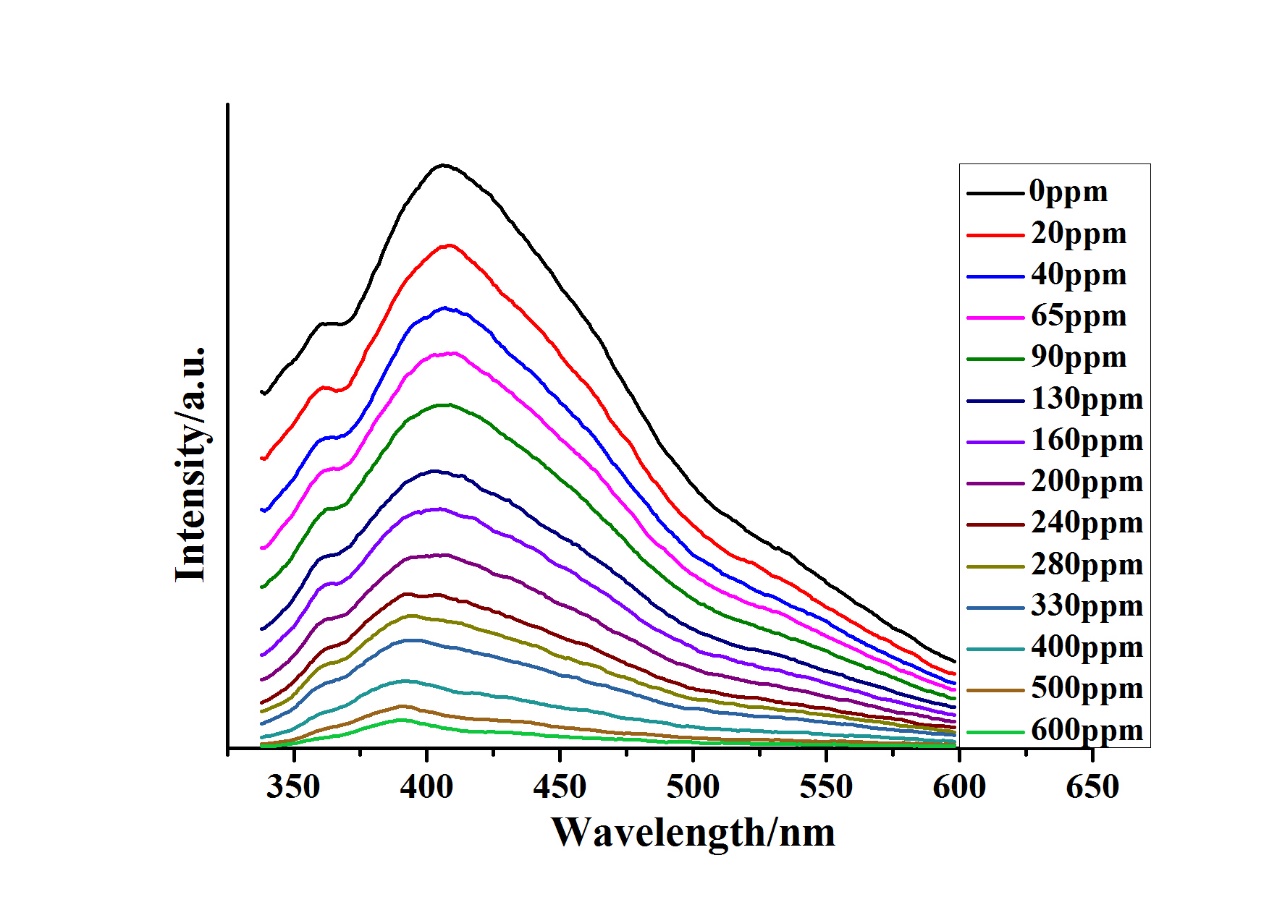


Fig. S16 Luminescent quenching of **1** dispersed in ethanol by the gradual addition of 1 mM solution of 4-NT in DMF.


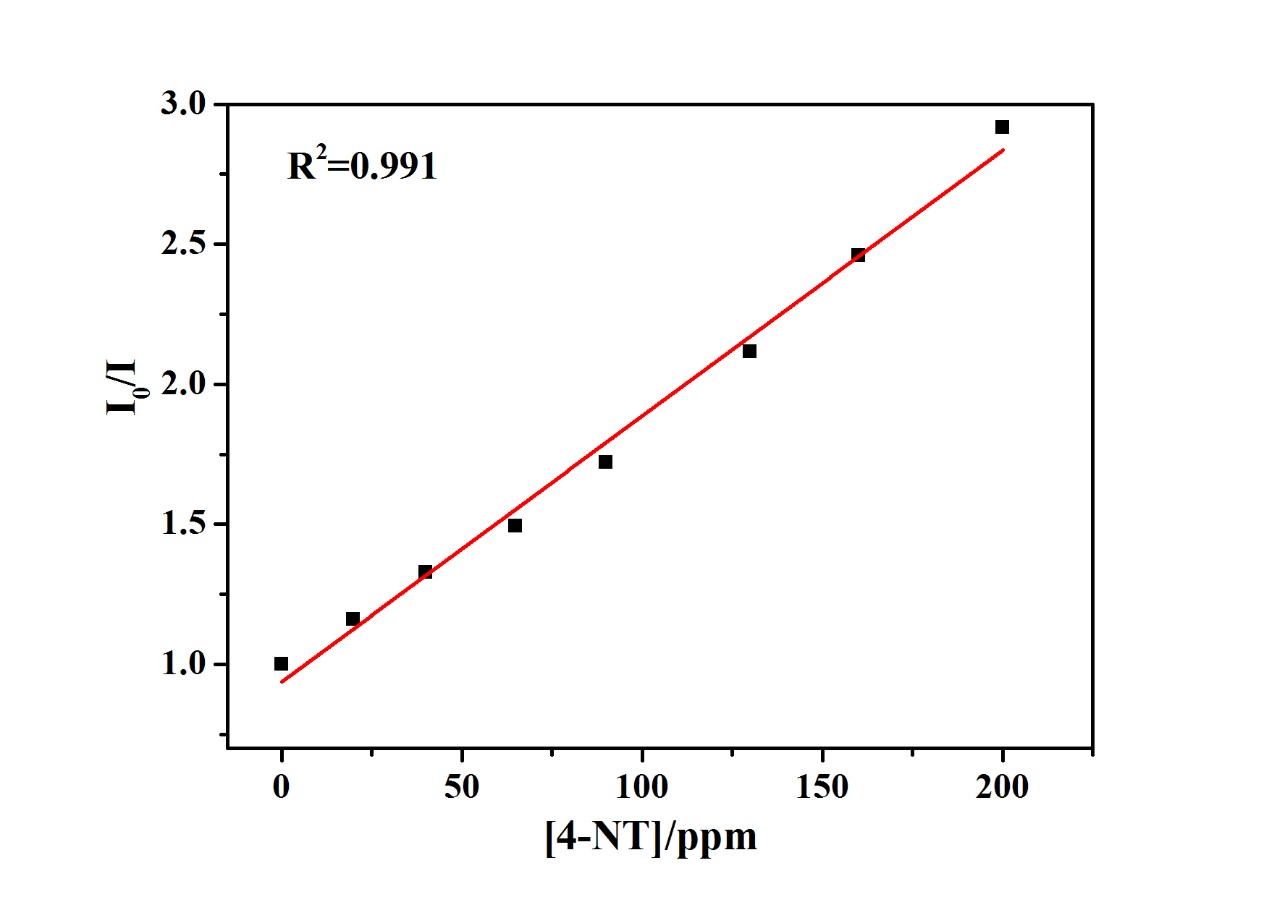


Fig. S17 Stern–Volmer plot for the fluorescence quenching of **1** upon the addition of 4-NT.


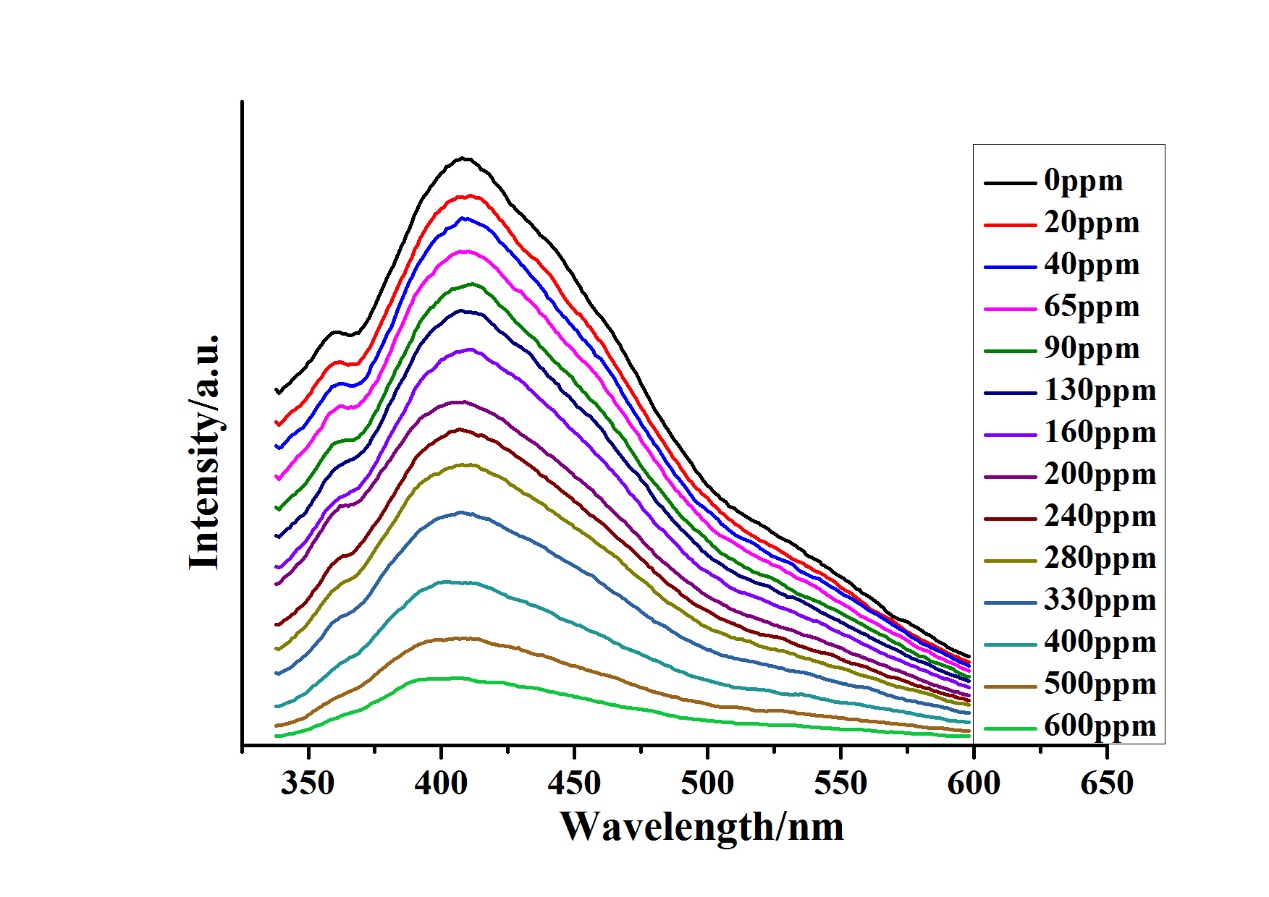


Fig. S18 Luminescent quenching of **1** dispersed in ethanol by the gradual addition of 1 mM solution of NB in DMF.


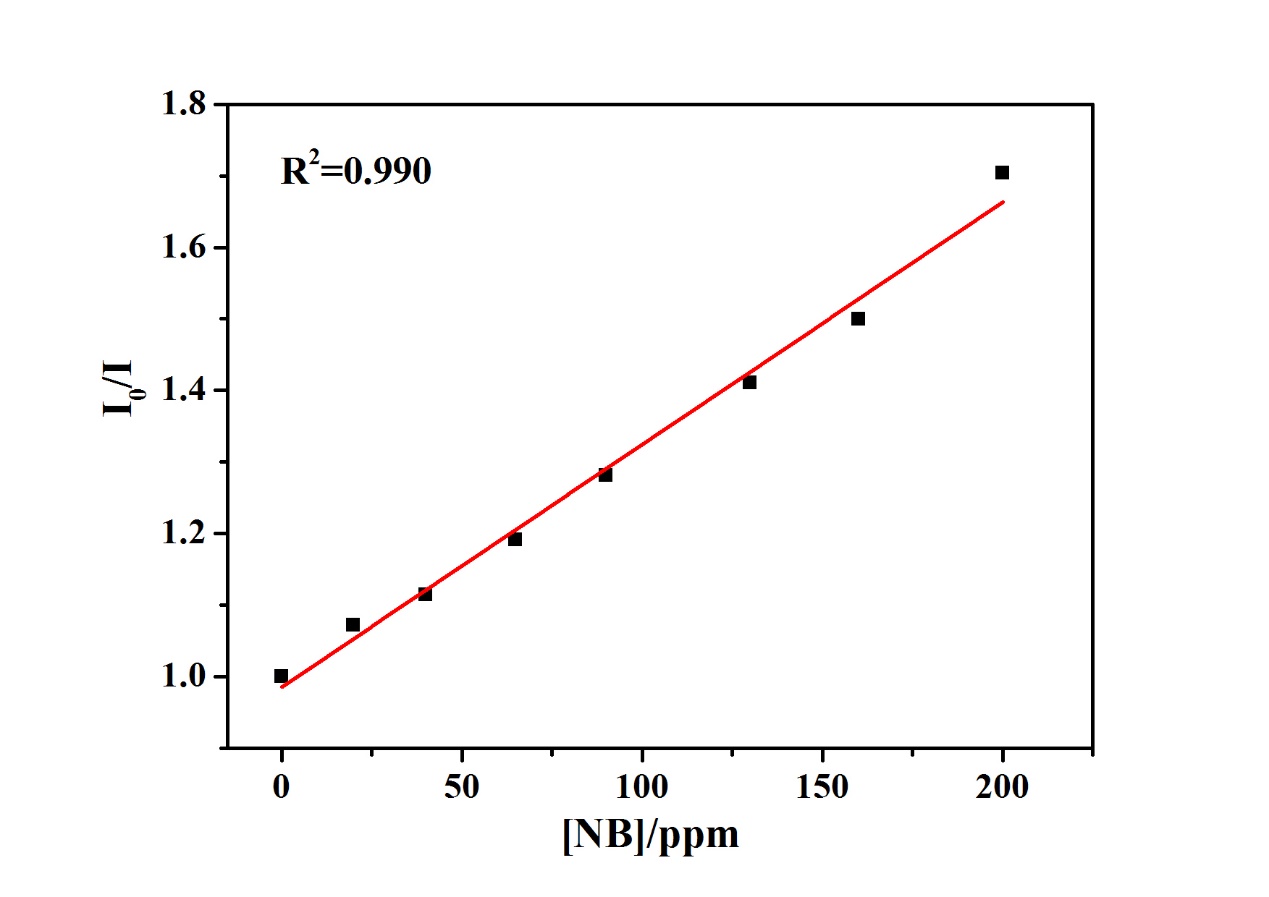


Fig. S19 Stern–Volmer plot for the fluorescence quenching of **1** upon the addition of NB.


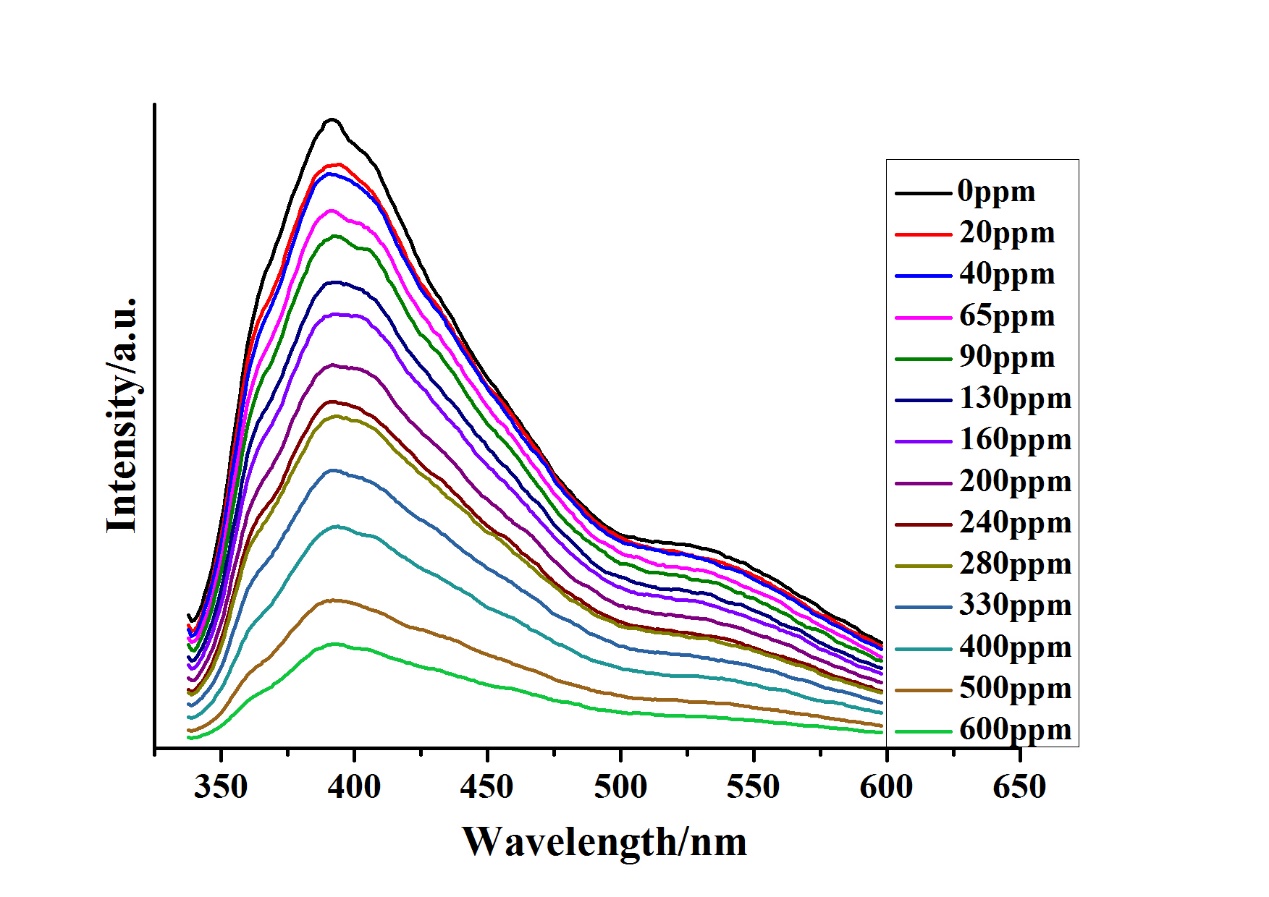


Fig. S20 Luminescent quenching of **2** dispersed in ethanol by the gradual addition of 1 mM solution of 1,3-DNB in DMF.


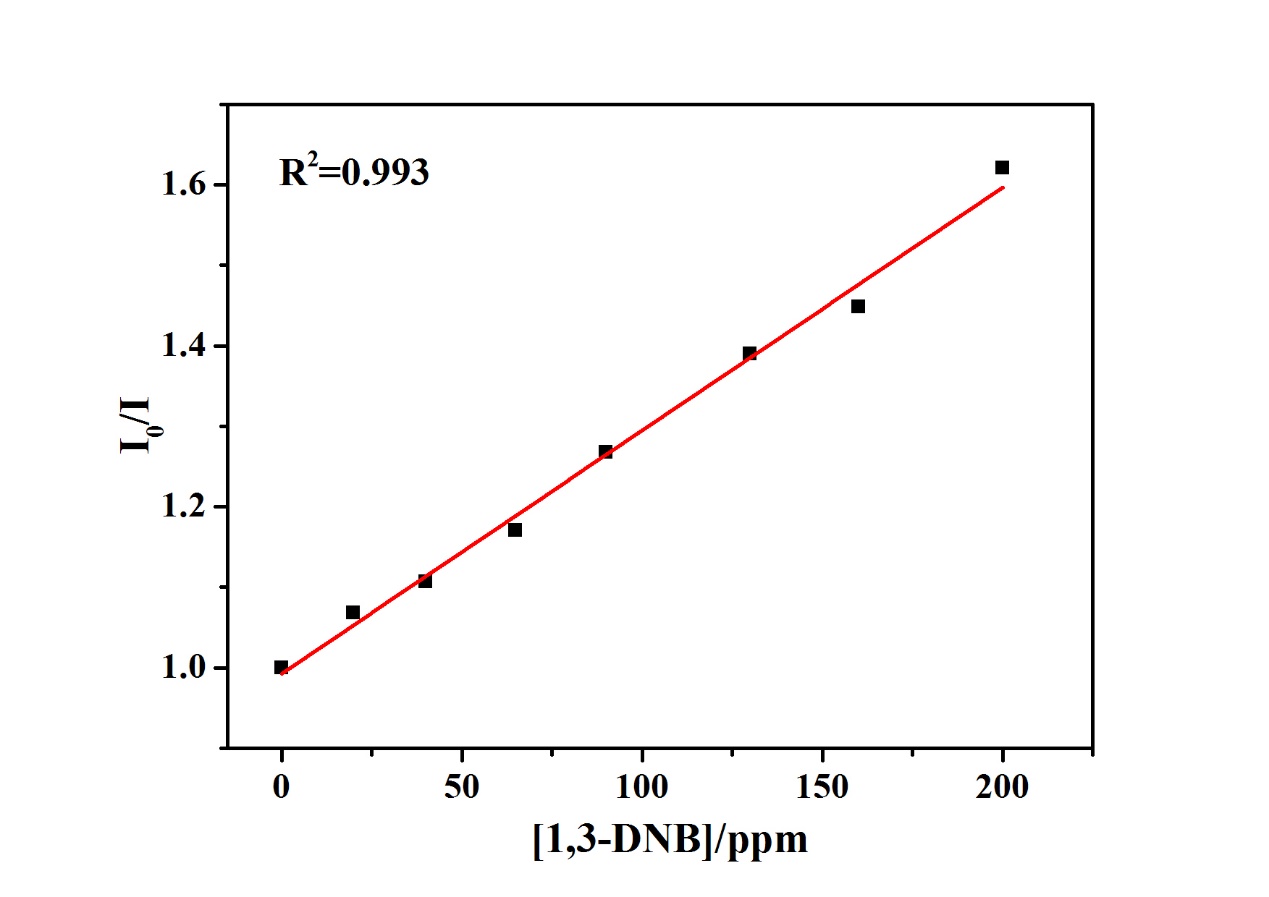


Fig. S21 The Stern–Volmer plot of **2** against 1,3-DNB.


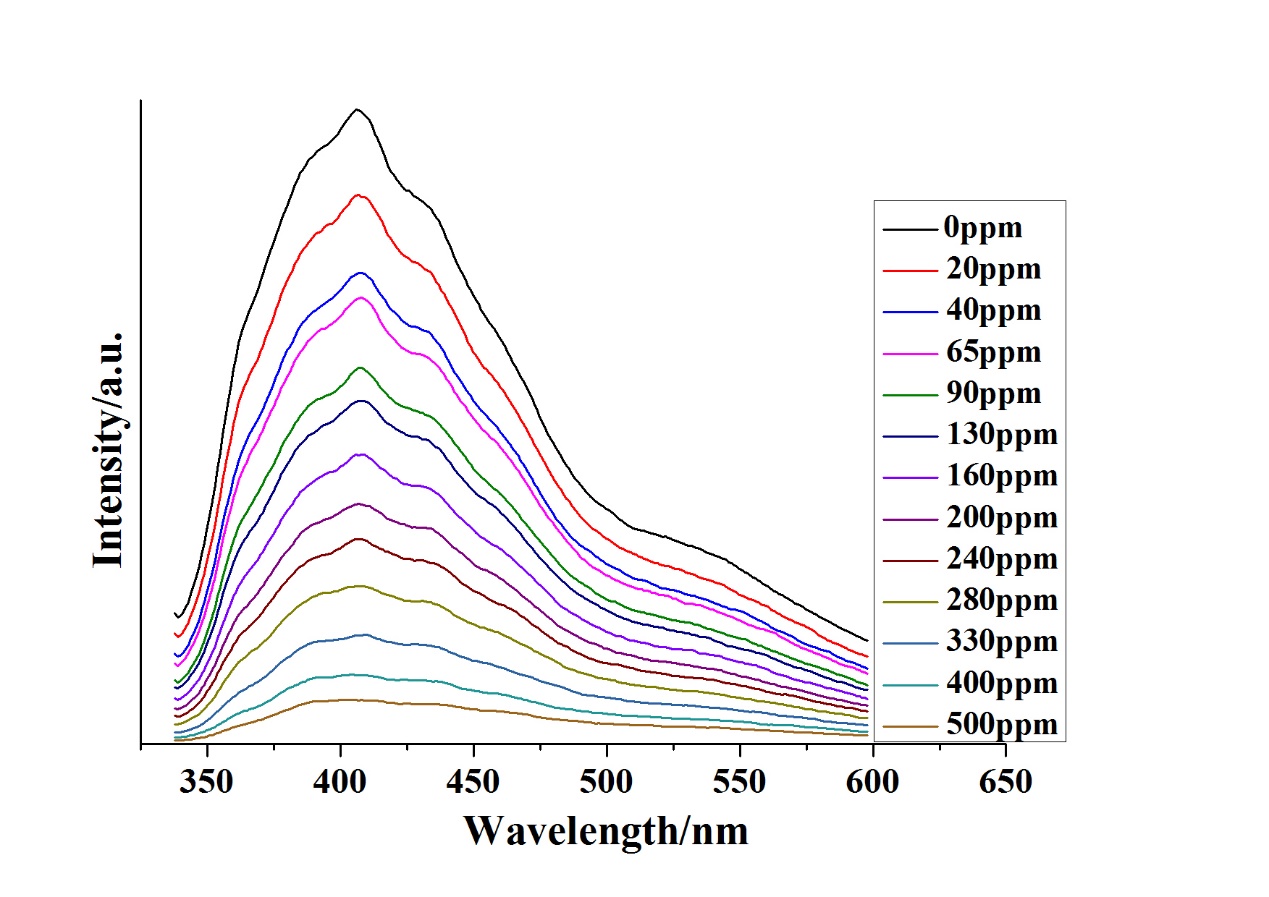


Fig. S22 Luminescent quenching of **2** dispersed in ethanol by the gradual addition of 1 mM solution of 2,4-DNT in DMF.


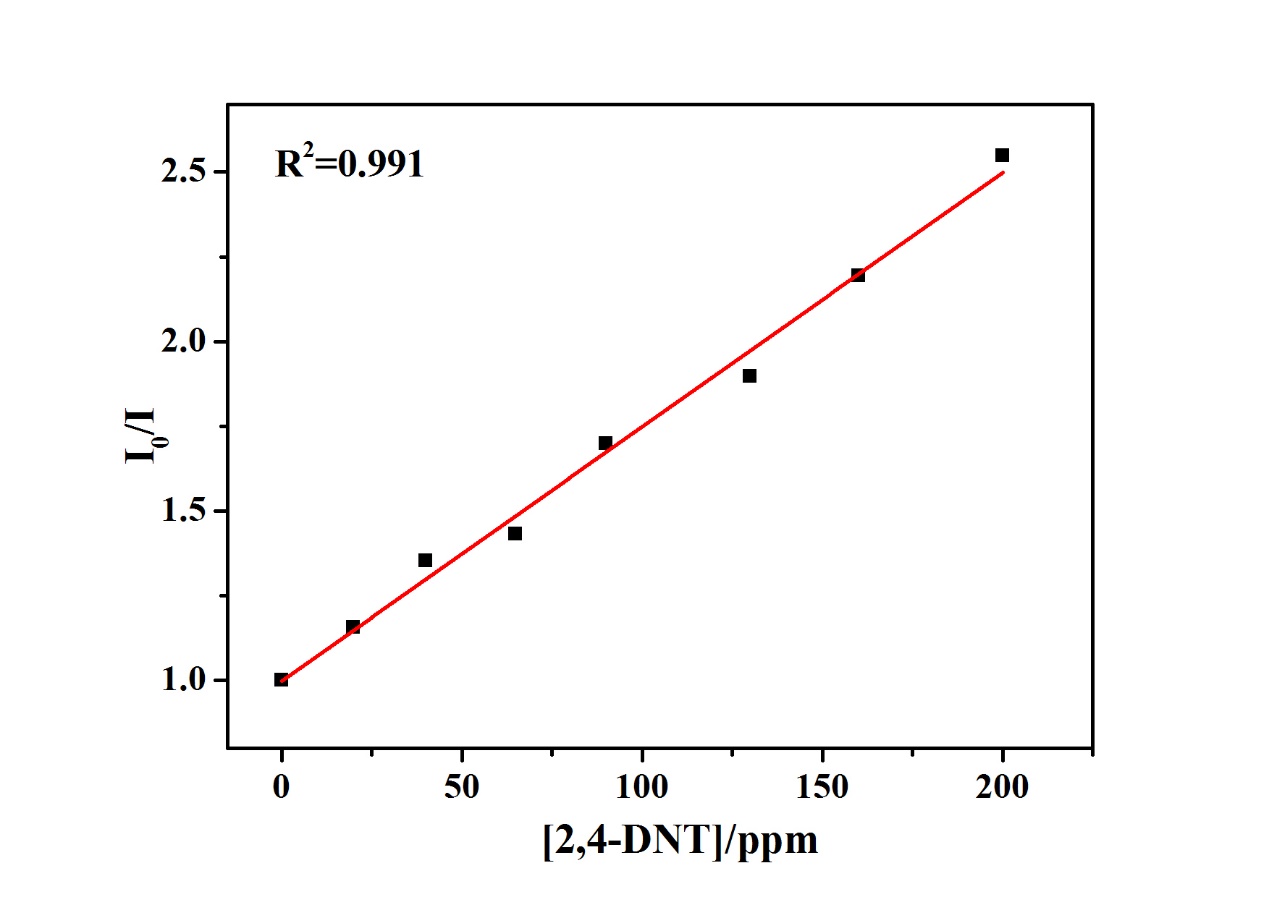


Fig. S23 Stern–Volmer plot for the fluorescence quenching of **2** upon the addition of 2,4-DNT.

**
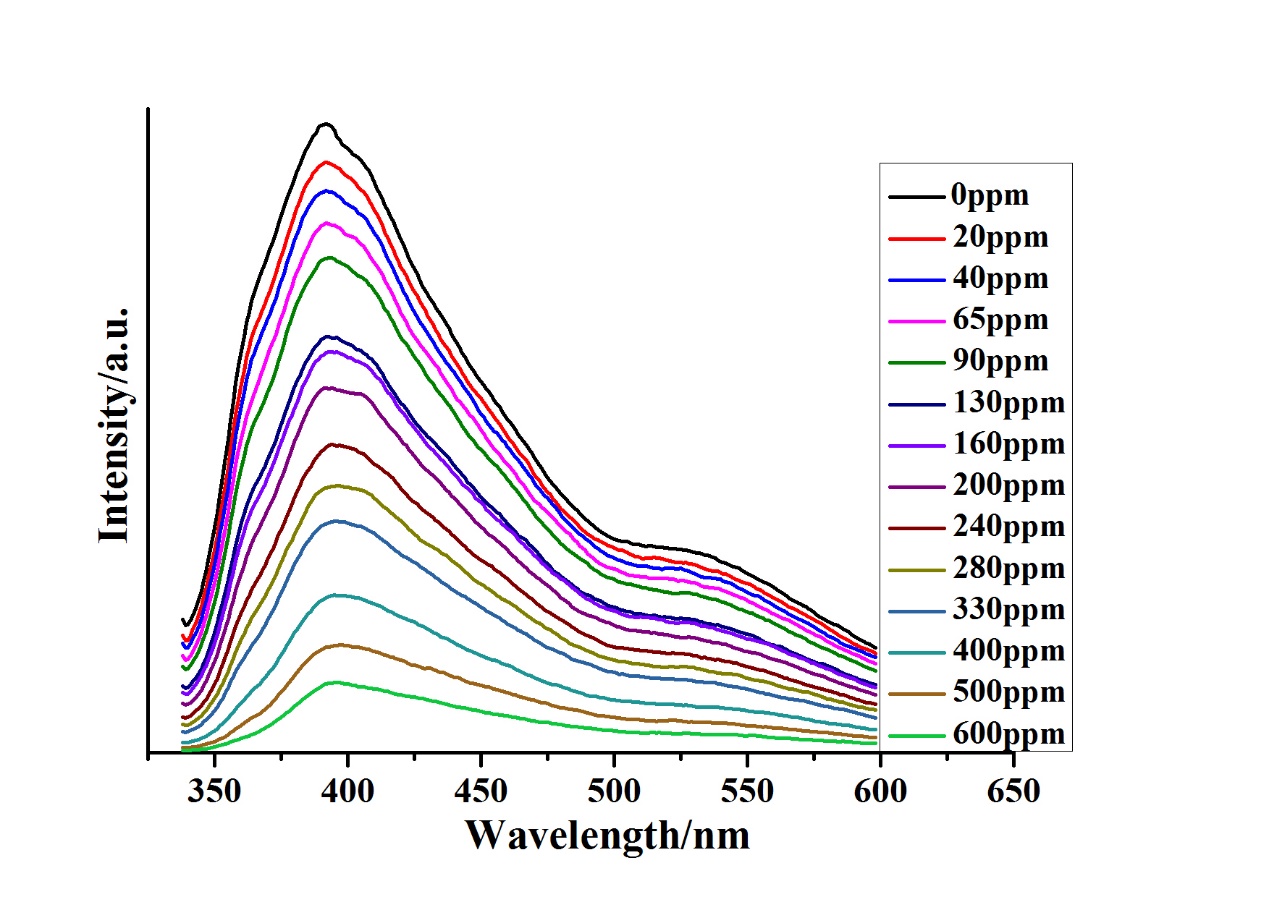
**

Fig. S24 Luminescent quenching of **2** dispersed in ethanol by the gradual addition of 1 mM solution of 2,6-DNT in DMF.


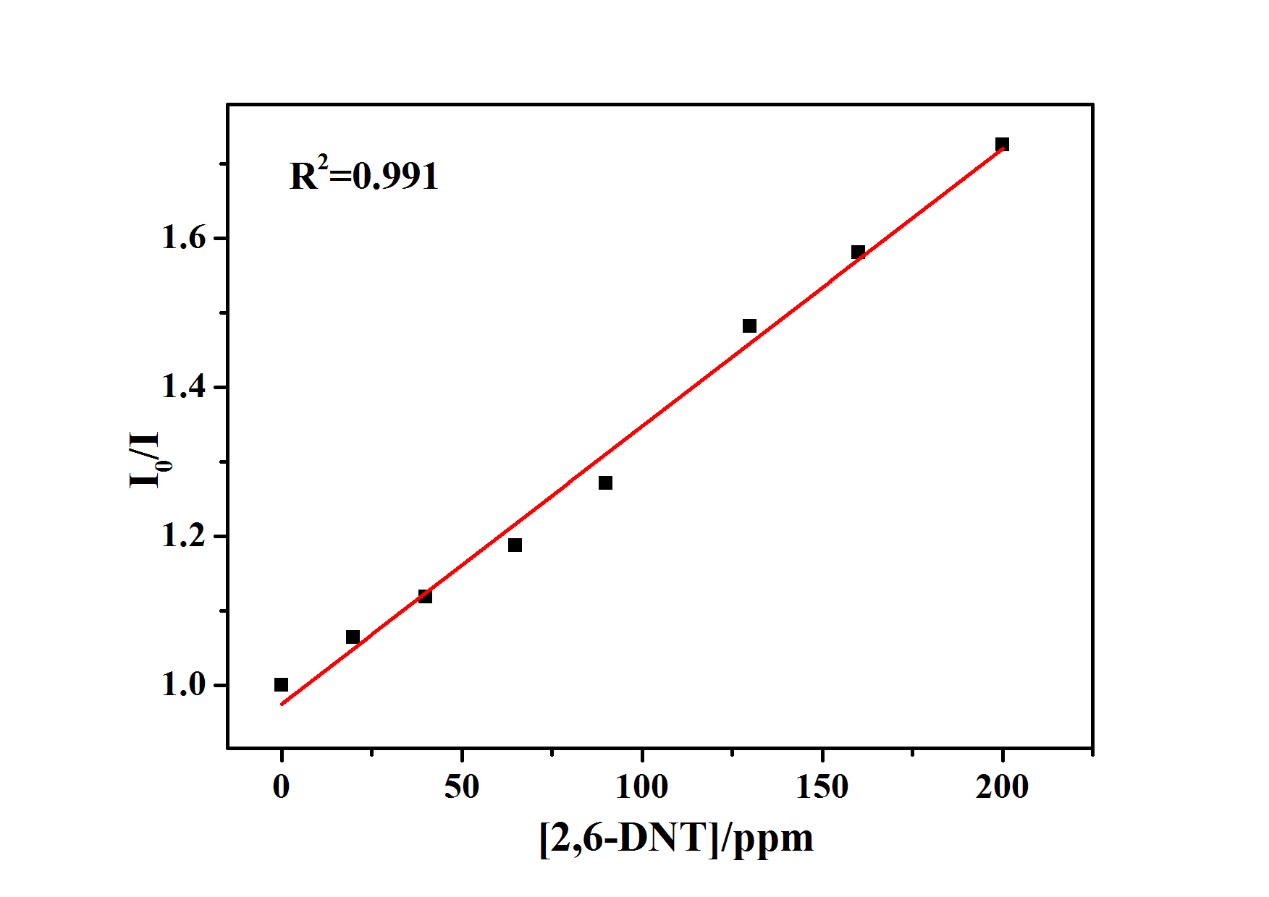


Fig. S25 Stern–Volmer plot for the fluorescence quenching of **2** upon the addition of 2,6-DNT.


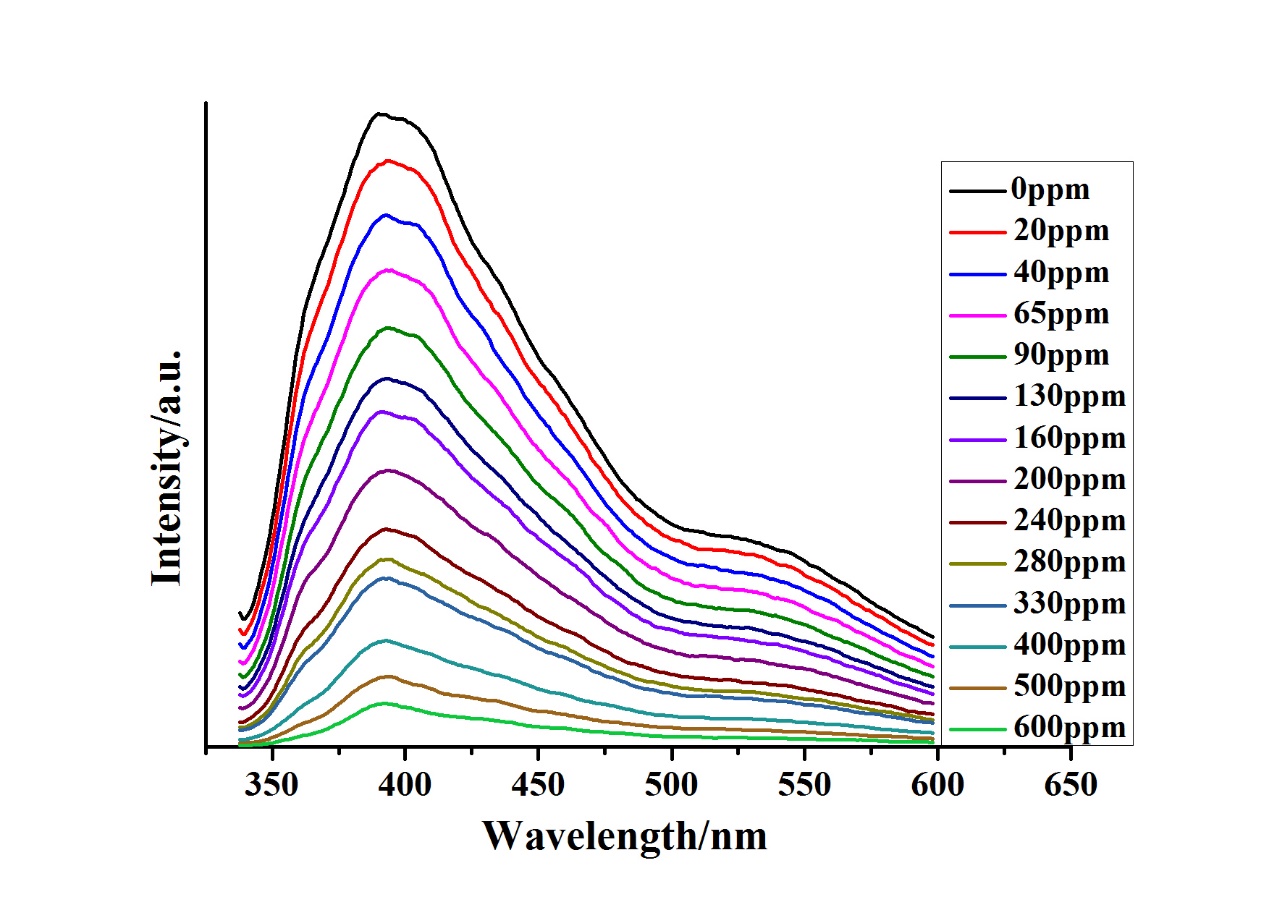


Fig. S26 Luminescent quenching of **2** dispersed in ethanol by the gradual addition of 1 mM solution of 2-NT in DMF.


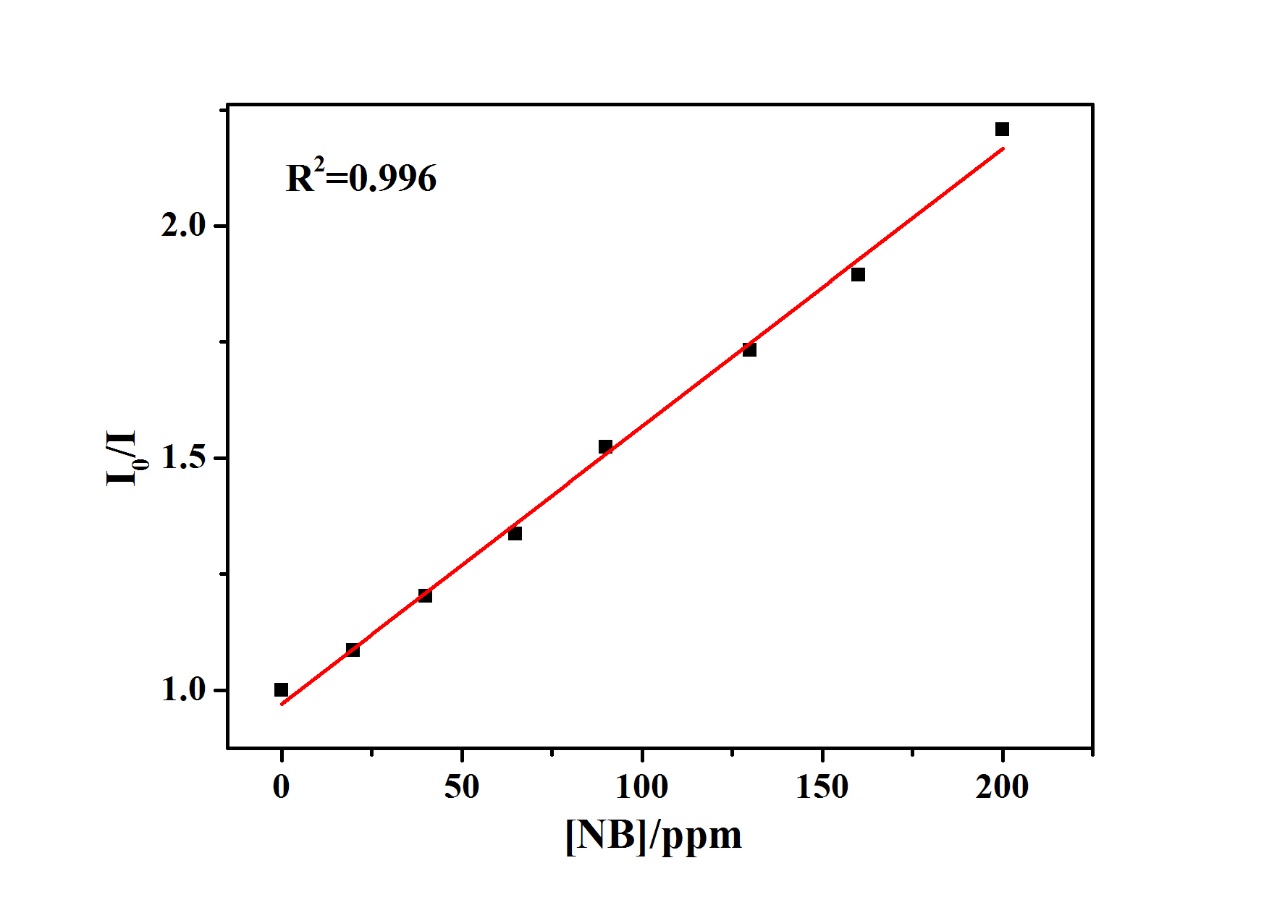


Fig. S27 Stern–Volmer plot for the fluorescence quenching of **2** upon the addition of 2-NT.


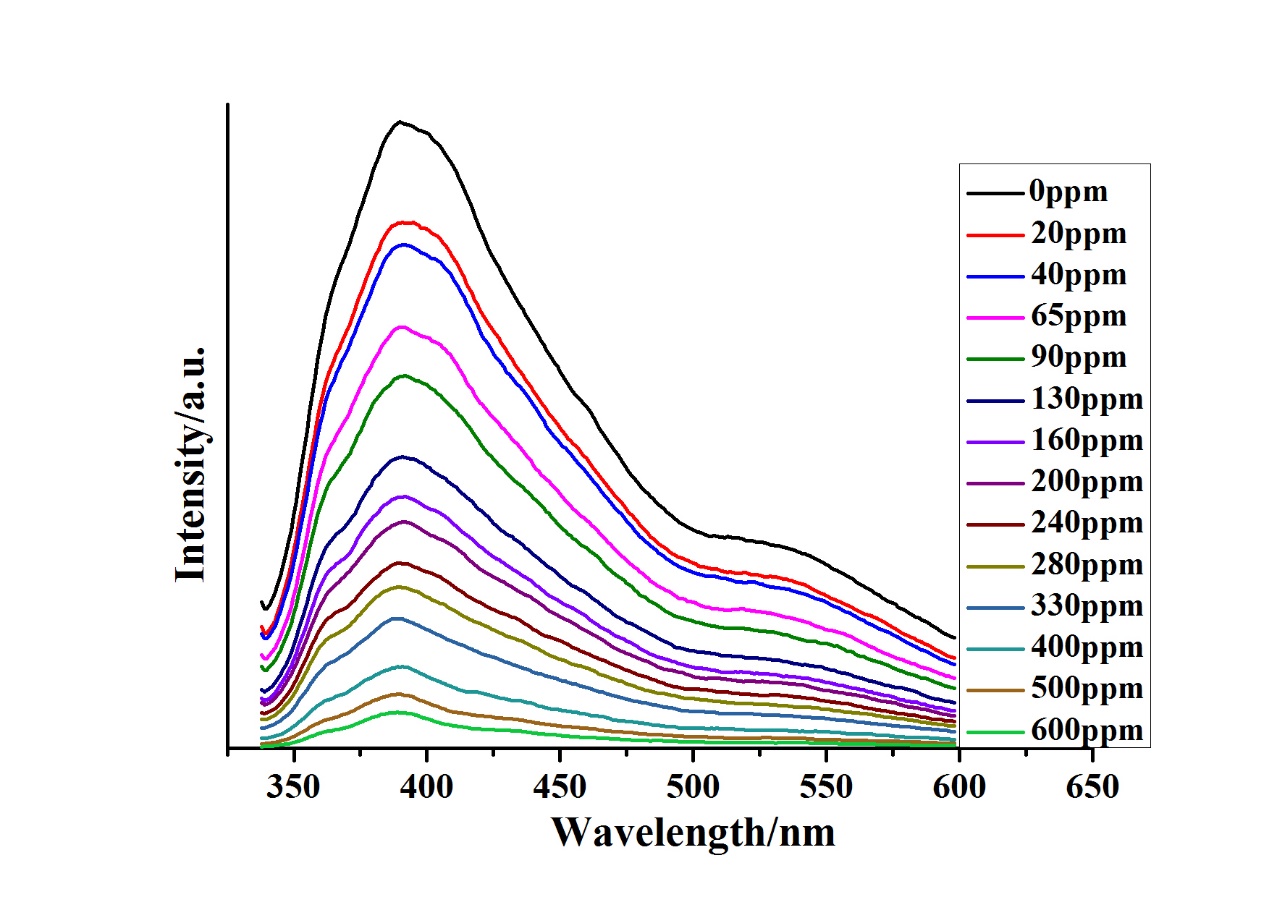


Fig. S28 Luminescent quenching of **2** dispersed in ethanol by the gradual addition of 1 mM solution of 4-NT in DMF.


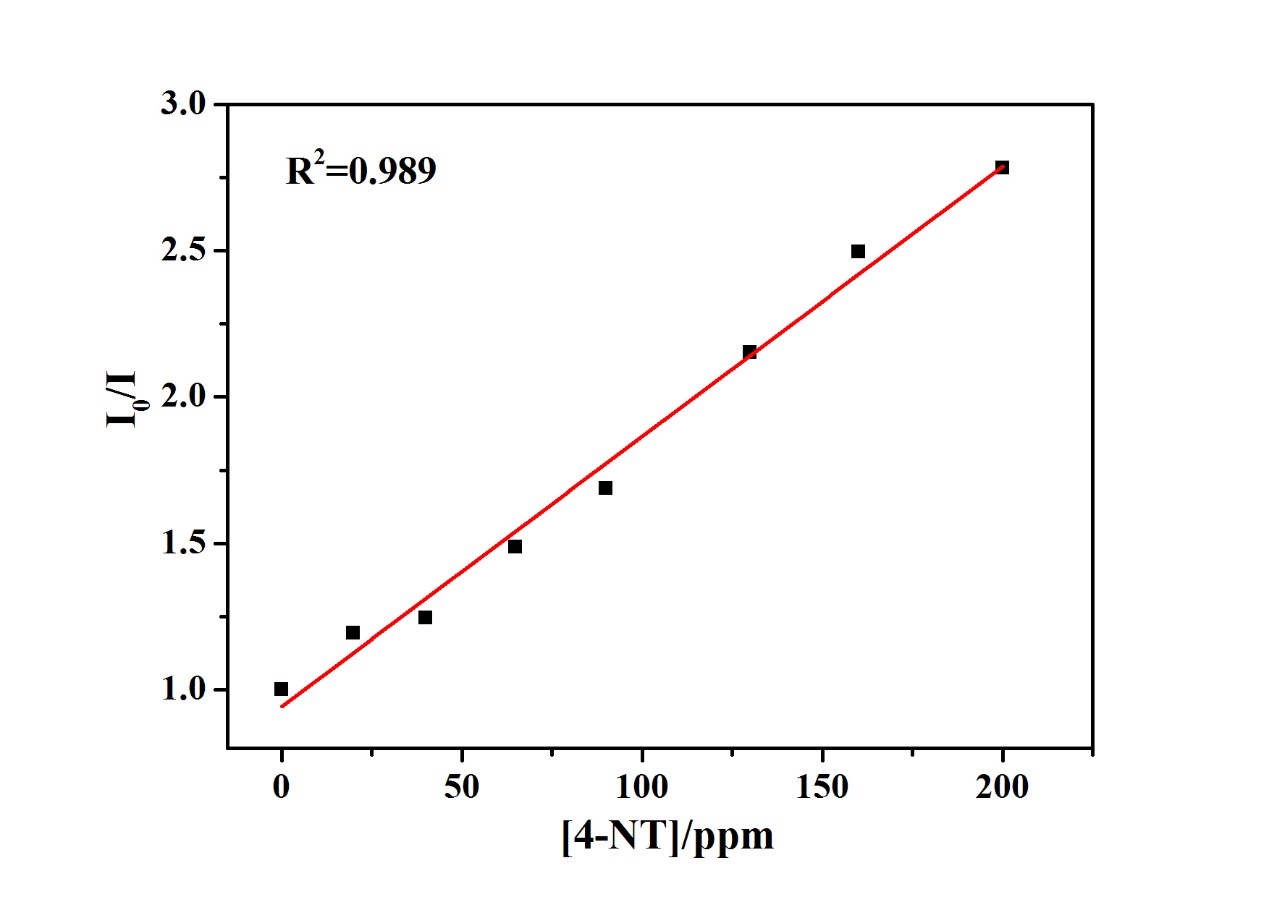


Fig. S29 Stern–Volmer plot for the fluorescence quenching of **2** upon the addition of 4-NT.


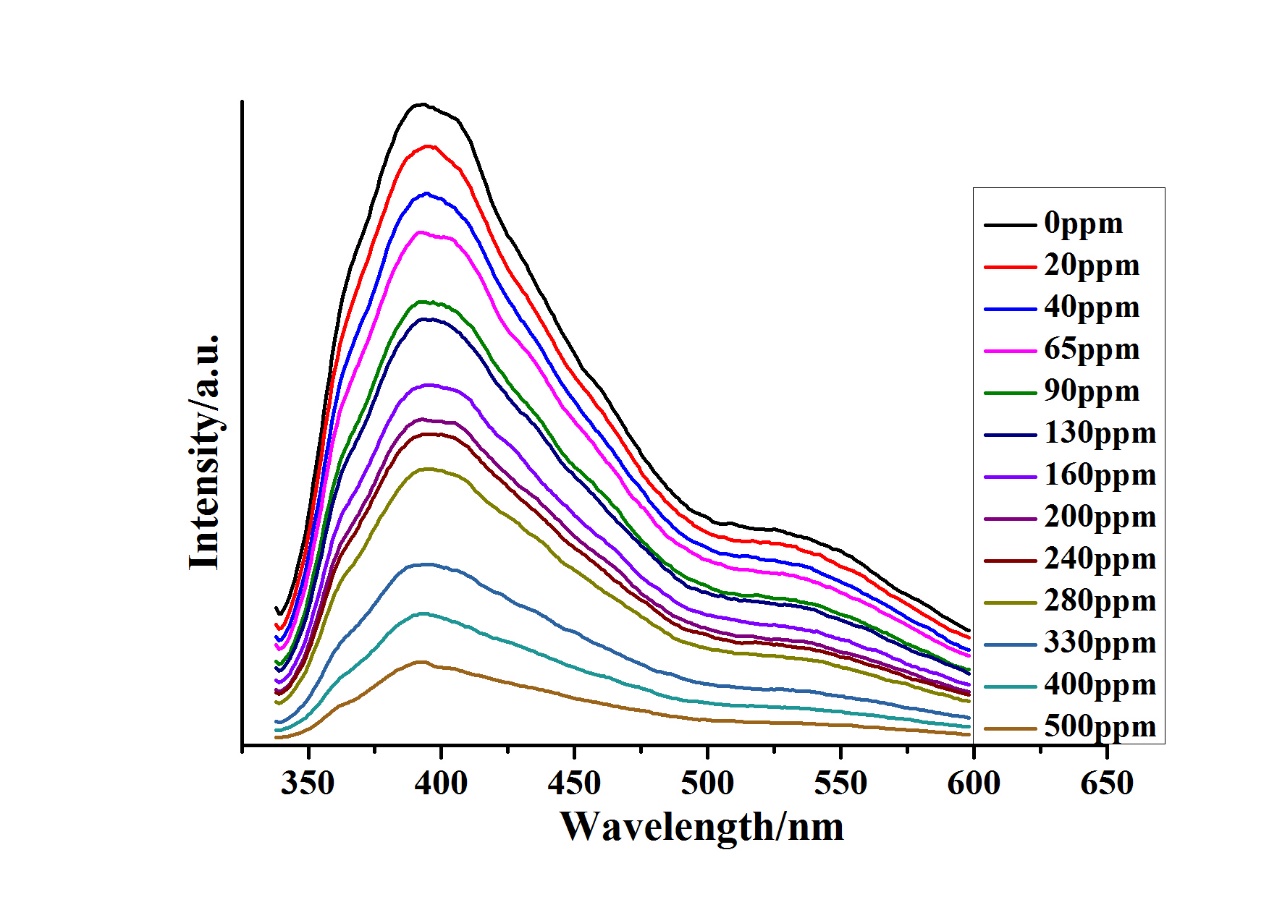


Fig. S30 Luminescent quenching of **2** dispersed in ethanol by the gradual addition of 1 mM solution of NB in DMF.


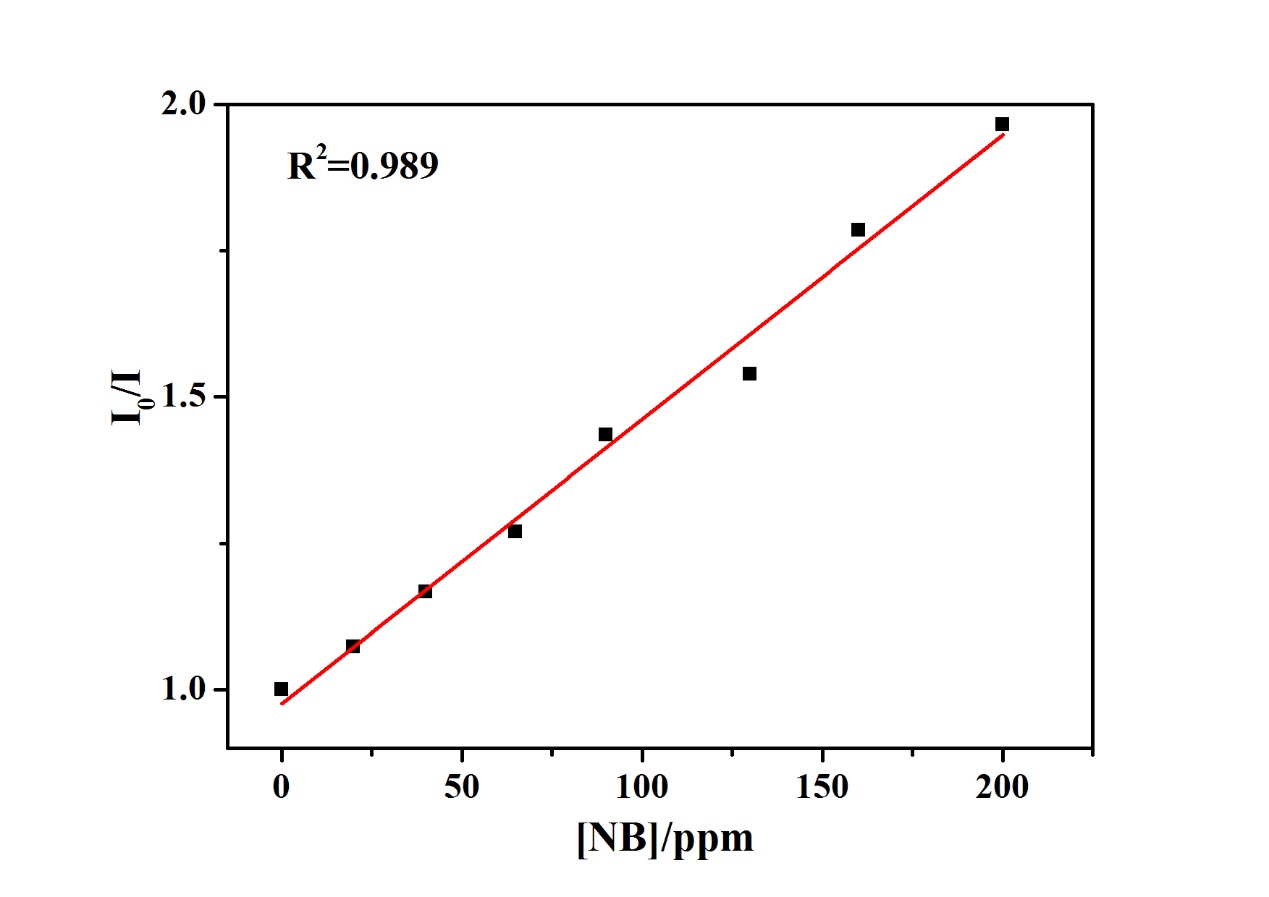


Fig. S31 Stern–Volmer plot for the fluorescence quenching of **1** upon the addition of NB.

Table S1 view of the fitting parameters in **1-2**

| **Sample** | | **1** | **2** |
| --- | --- | --- | --- |
| **Fe(NO_3_)_3_** | **Ksv** | **8.59×10^3^** | **8.14×10^3^** |
|  | **LOD** | **0.75** | **0.79** |
| **NB** | **Ksv** | **4.17×10^2^** | **5.98×10^2^** |
|  | **LOD** | **1.88** | **1.81** |
| **2-NT** | **Ksv** | **8.67×10^2^** | **8.20×10^2^** |
|  | **LOD** | **1.63** | **1.68** |
| **4-NT** | **Ksv** | **1.30×10^3^** | **1.26×10^3^** |
|  | **LOD** | **1.12** | **1.17** |
| **2,4-DNT** | **Ksv** | **1.31×10^3^** | **1.37×10^3^** |
|  | **LOD** | **1.37** | **1.33** |
| **2,6-DNT** | **Ksv** | **9.03×10^2^** | **6.78×10^2^** |
|  | **LOD** | **1.78** | **1.89** |
| **1,3-DNB** | **Ksv** | **5.83×10^2^** | **5.08×10^2^** |
|  | **LOD** | **1.74** | **1.83** |
| **TNP** | **Ksv** | **2.85×10^3^** | **2.25×10^3^** |
|  | **LOD** | **0.86** | **0.94** |


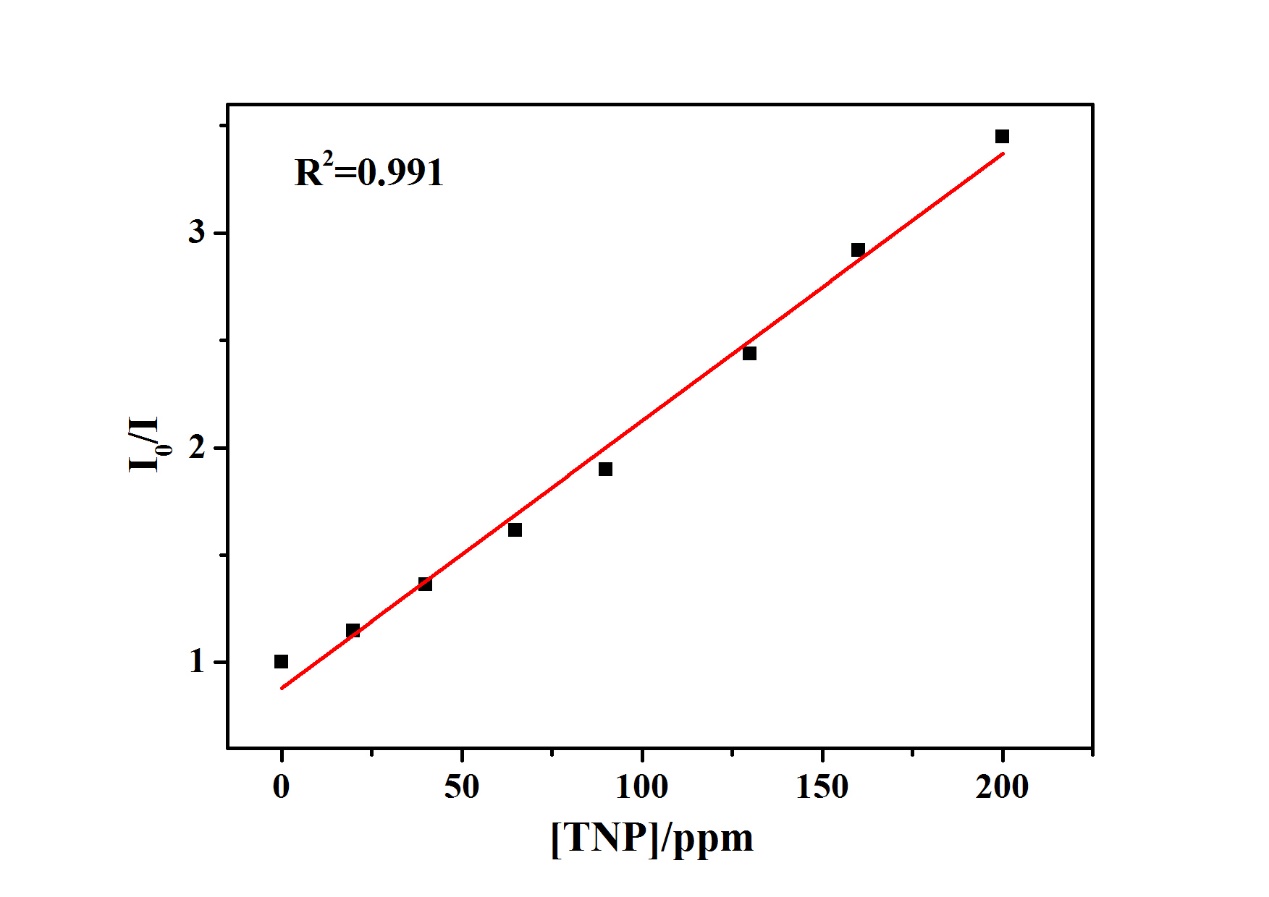


Fig. S32 Stern–Volmer plot for the fluorescence quenching of **1** upon the addition of TNP.


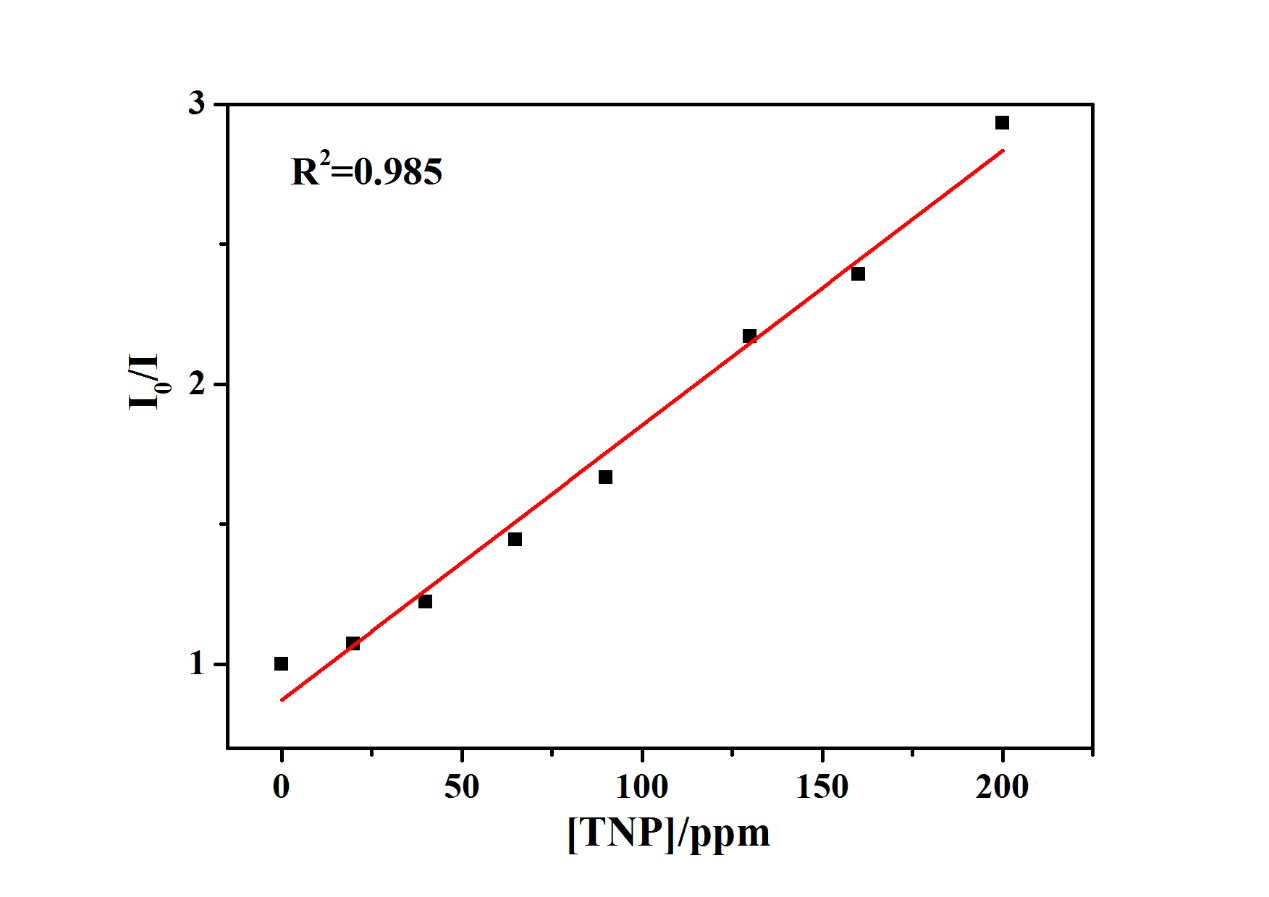


Fig. S33 Stern–Volmer plot for the fluorescence quenching of **2** upon the addition of TNP.

| 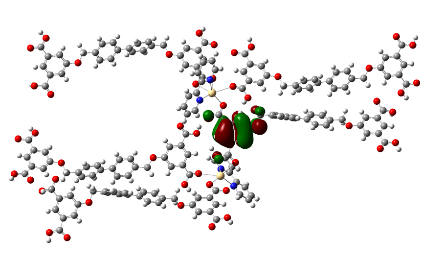  HOMO **1** | 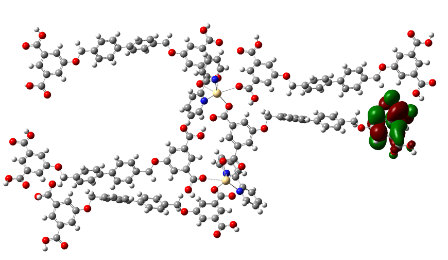  LUMO **1** |
| --- | --- |
| 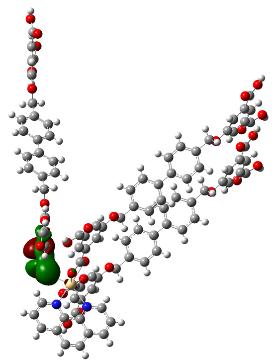  HOMO **2** | 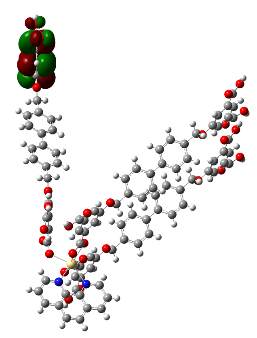  LUMO **2** |
| 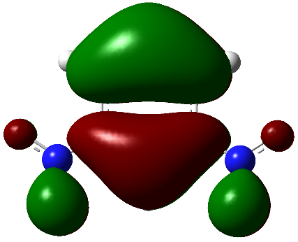  HOMO 1,3-DNB | 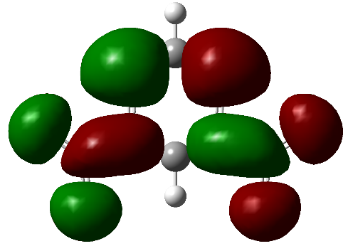  LUMO 1,3-DNB |
| 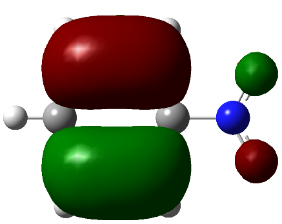  HOMO NB | 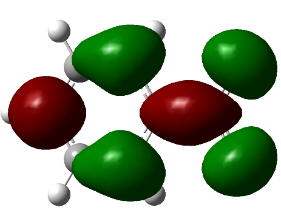  LUMO NB |
| 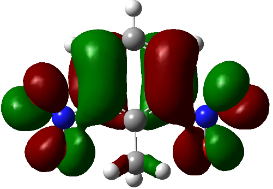  HOMO 2,6-DNT | 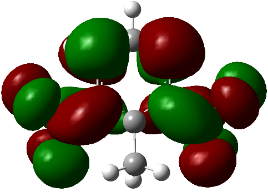  LUMO 2,6-DNT |
| 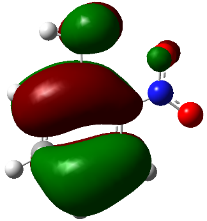  HOMO 2-NT | 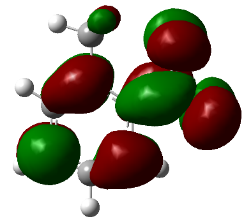  LUMO 2-NT |

Figure S34a HOMO–LUMO plots of the NACs along with CPs **1** and **2**.

| 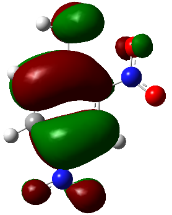  HOMO 2,4-DNT | 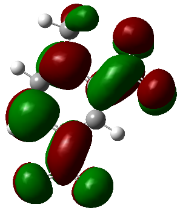  LUMO 2,4-DNT |
| --- | --- |
| 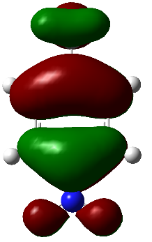  HOMO 4-NT | 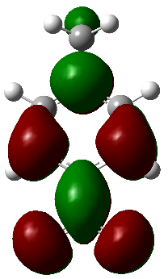  LUMO 4-NT |
| 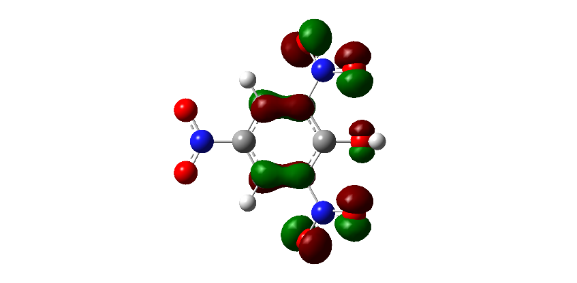  HOMO TNP | 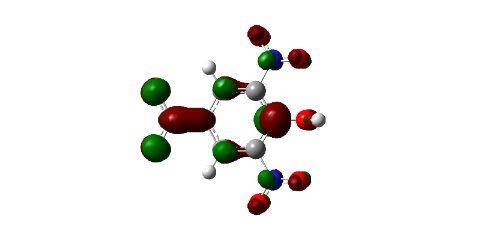  LUMO TNP |
| 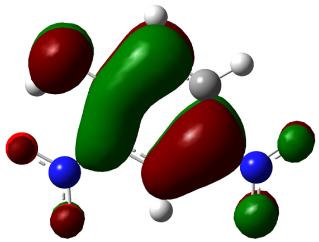  HOMO 2,4-DNP | 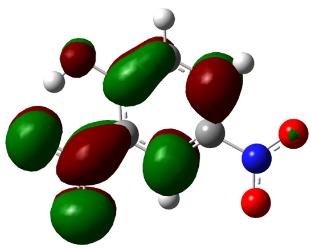  LUMO 2,4-DNP |
| 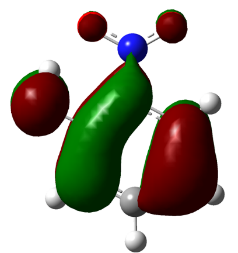  HOMO o-nitro phenol | 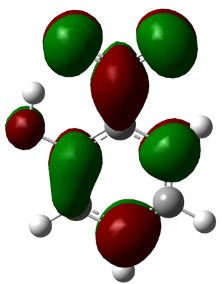  LUMO o-nitro phenol |
| 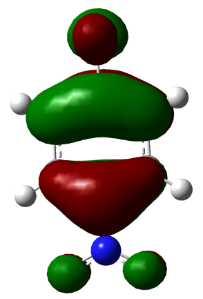  HOMO p-nitrophenol | 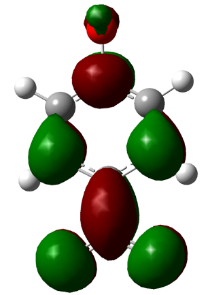  LUMO p-nitrophenol |

Figure S34b HOMO–LUMO plots of the NACs.


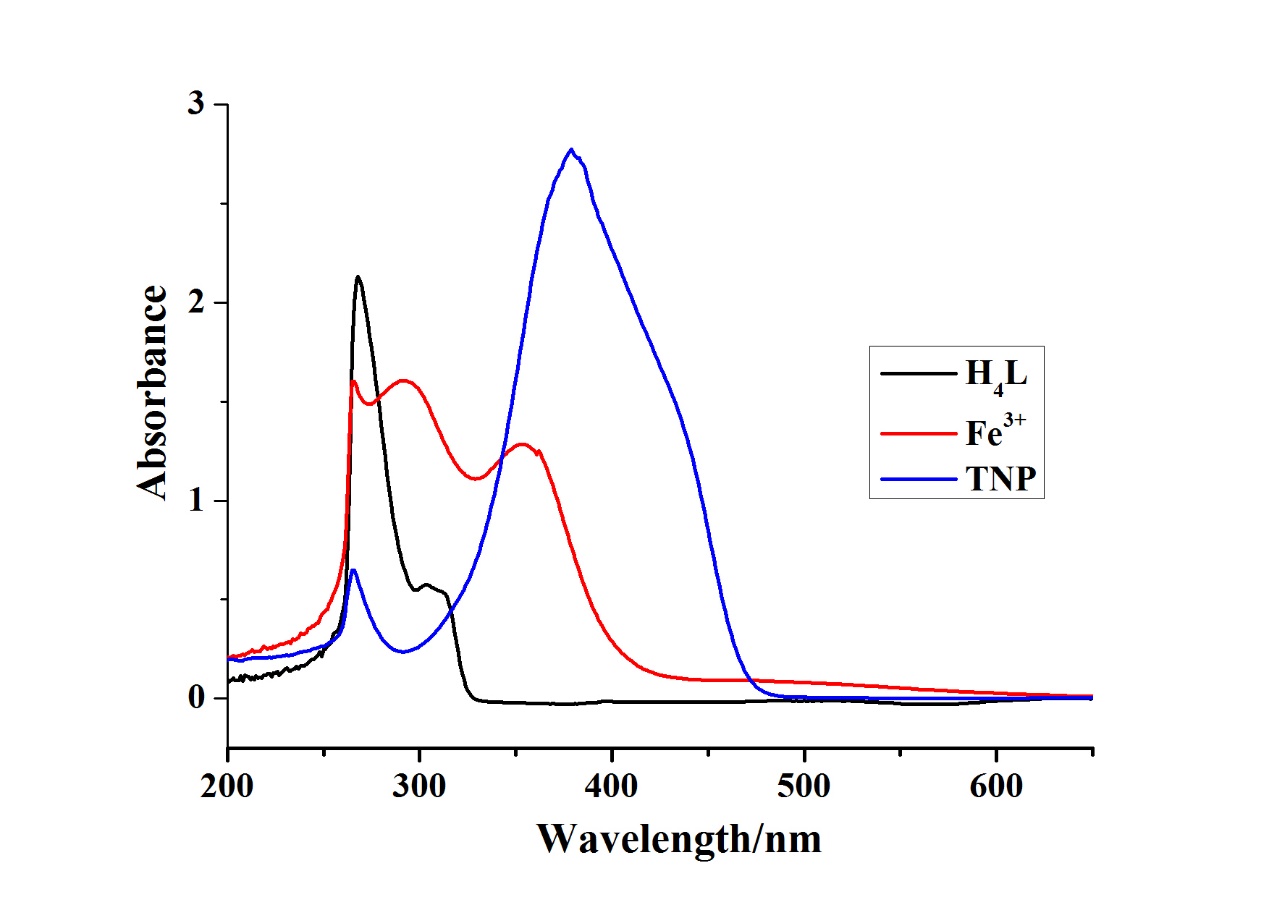


Fig. S35 UV-Vis absorption spectra.


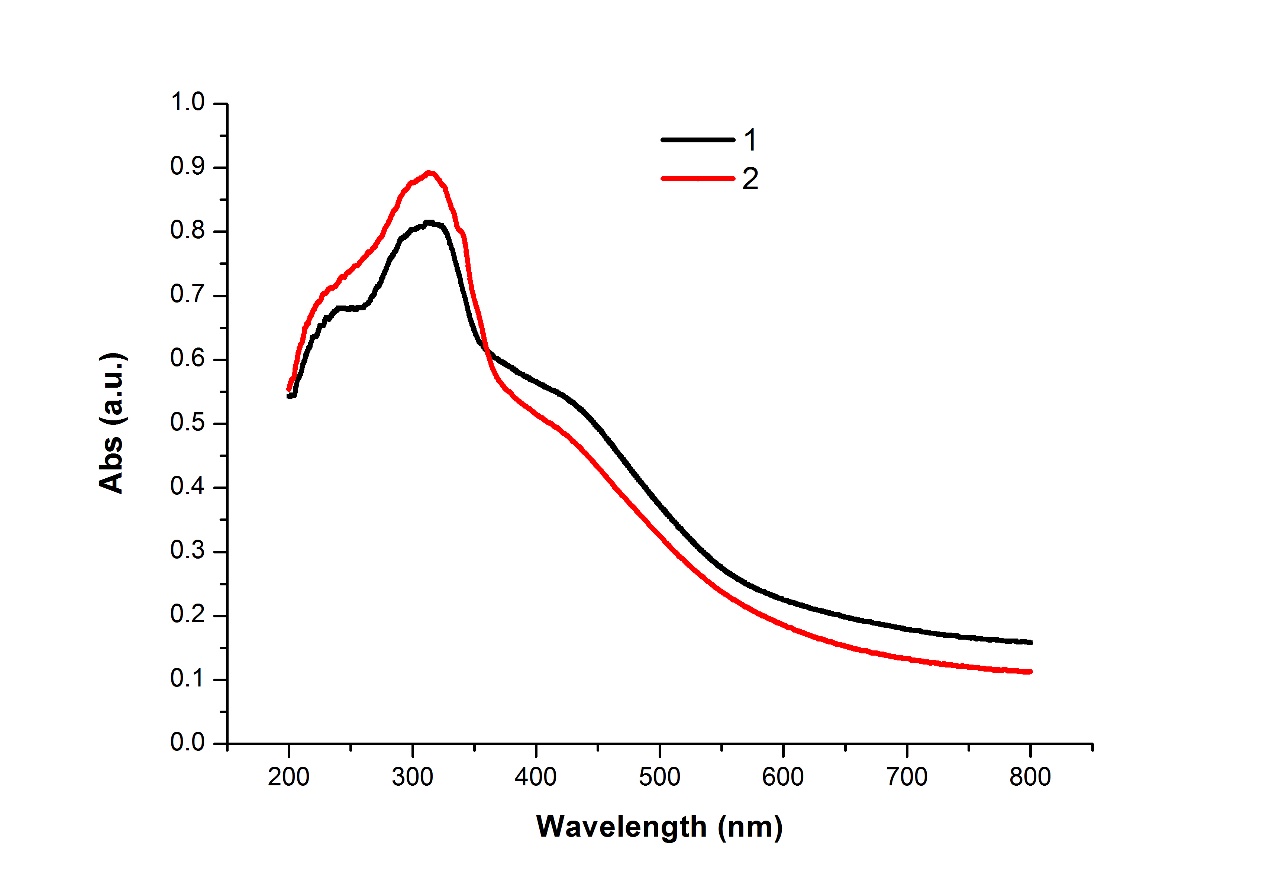


Fig. S36. UV–vis diffuse-reflectance spectra of **1-2** with BaSO_4_ as background.


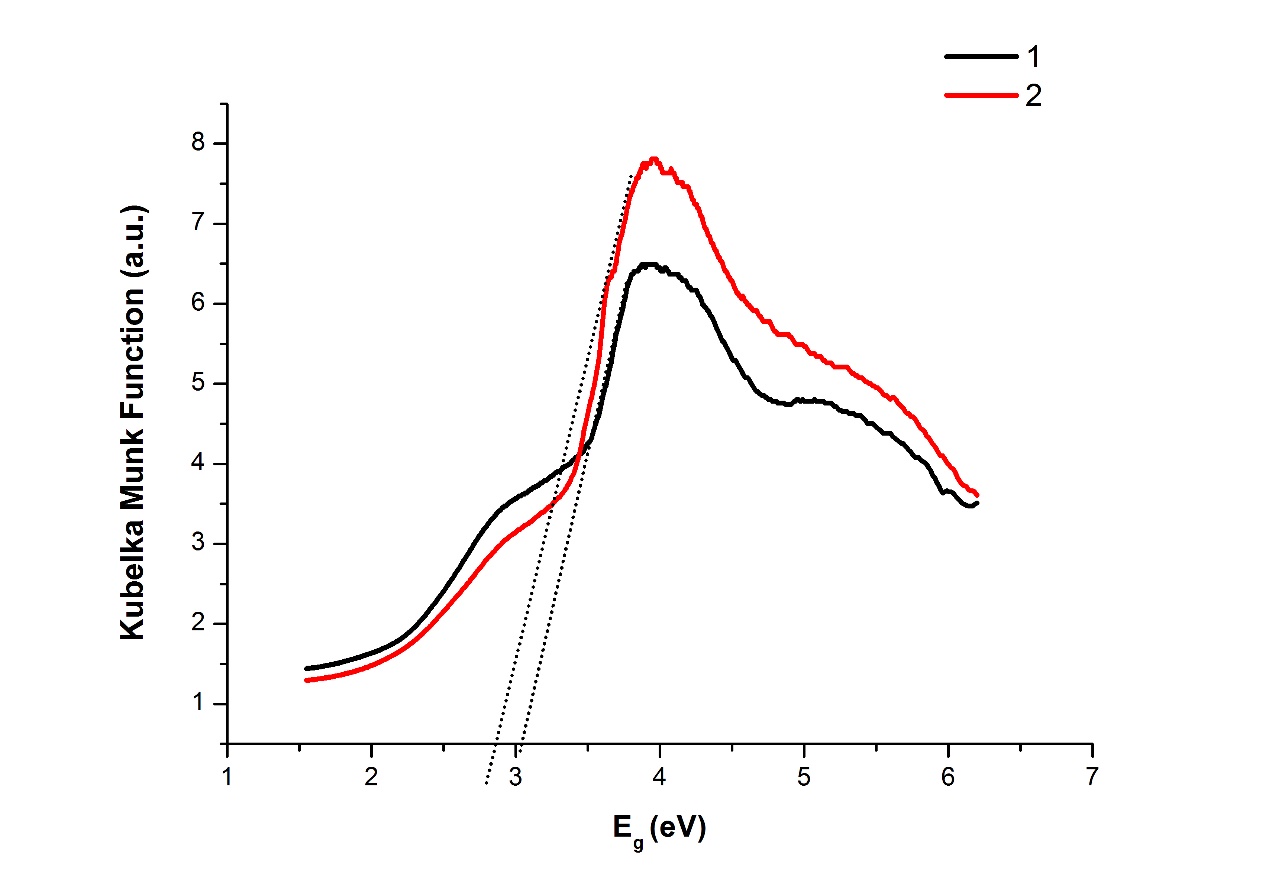


Fig. S37 Solid-state optical diffuse-reflection spectra of **1-2** derived from diffuse reflectance data at ambient temperature. The intercept of the extrapolated absorption edge on the energy scale (x axis) gives the band gap of the sample.


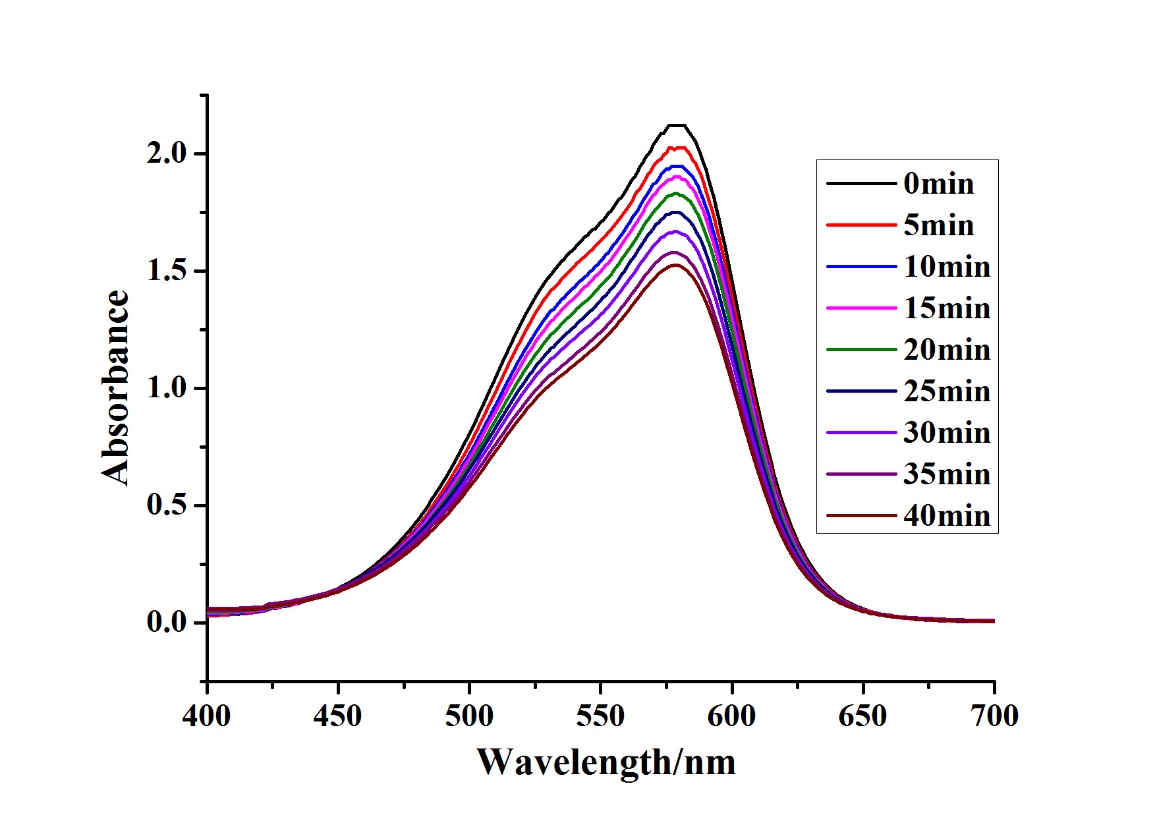


Fig. S38 UV–vis absorption spectra of the MV solution during the decomposition reaction under 250W Hg lamp irradiation.


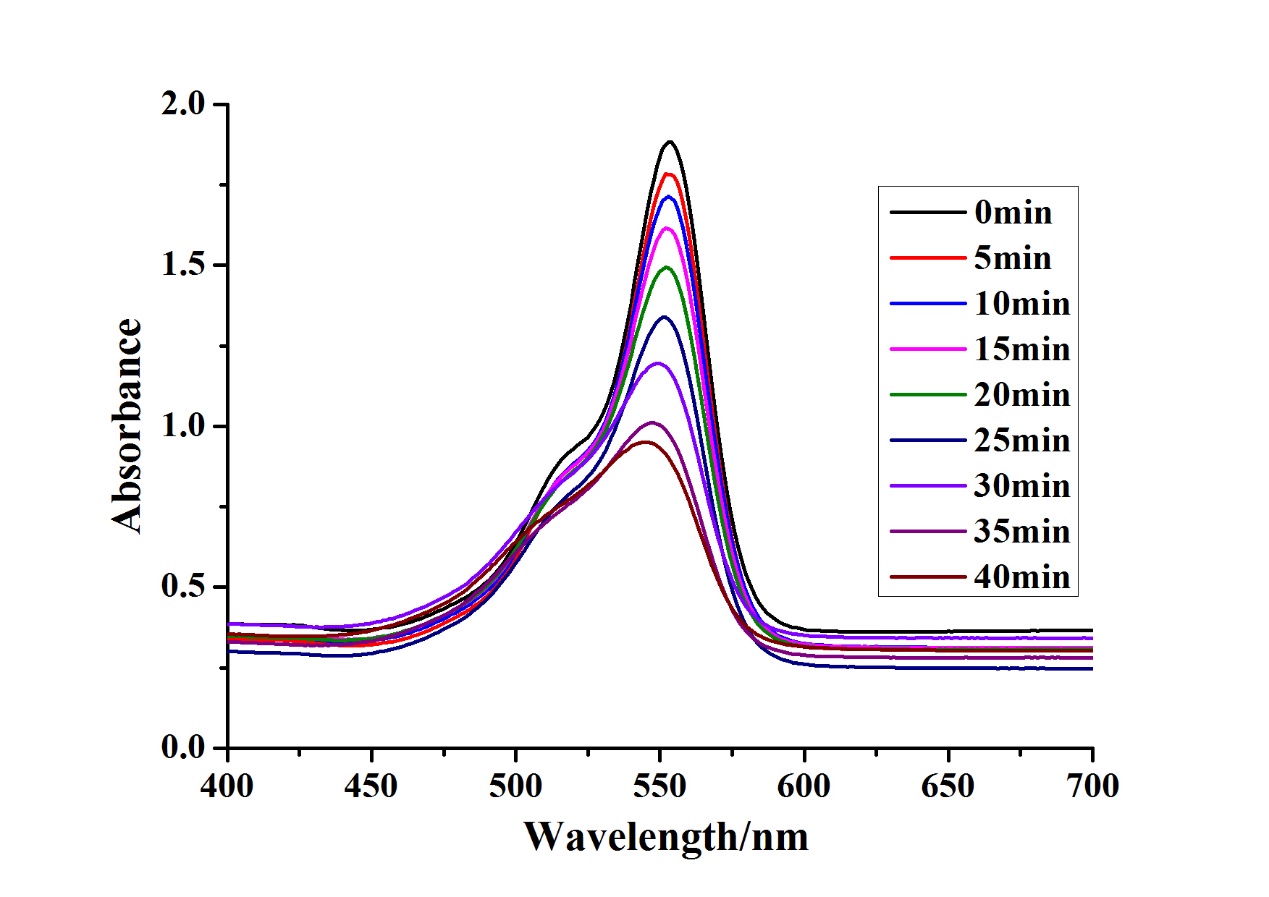


Fig. S39 UV–vis absorption spectra of the Rh B solution during the decomposition reaction under 250W Hg lamp irradiation.


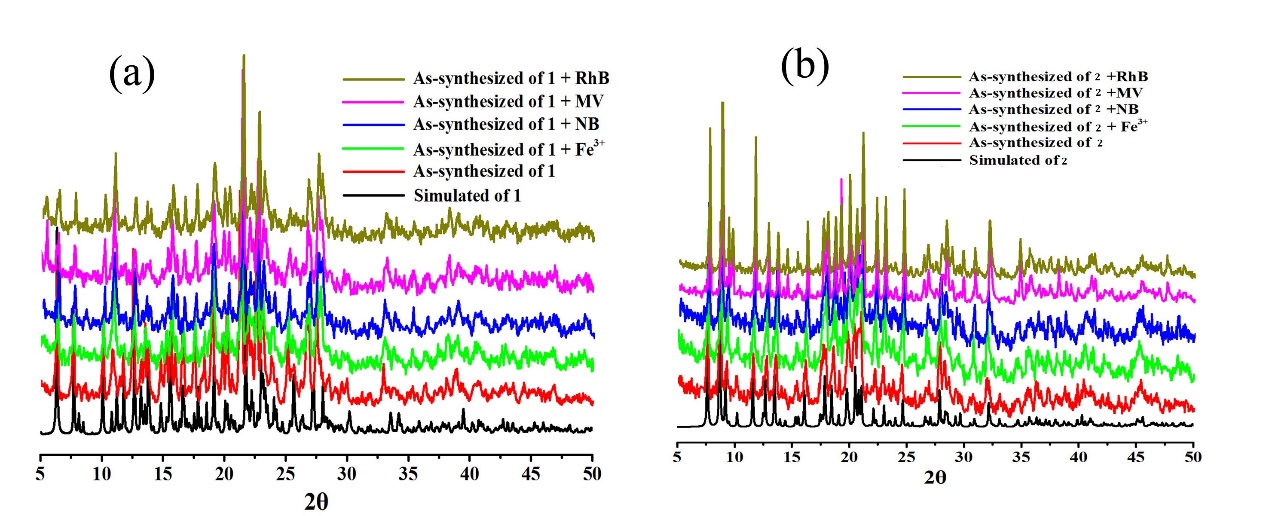


Fig. 40 the PXRD pattern for different incorporated inclusions in the **1** and **2.**

**Table S2. Crystallographic data and structure refinement details for Complexes 1-2**

| Parameter | **1** | **2** |
| --- | --- | --- |
| Formula weight | 1616.09 | 1141.69 |
| Crystal system | Monoclinic | Monoclinic |
| Space group | *P2_1_/c* | *P2/c* |
| Crystal Cdlor | Yellow | Colorless |
| *a*, Å | 8.0775(16) | 9.9883(10) |
| *b*, Å | 35.055(7) | 10.1533(10) |
| *c*, Å | 24.056(6) | 24.0657(19) |
| *α*, ° | 90 | 90 |
| *β*, ° | 108.36(3) | 105.898(3) |
| *γ*, ° | 90 | 90 |
| *V*, Å ^3^ | 6465(3) | 2347.3(4) |
| *Z* | 4 | 2 |
| ρ_calcd_, g/cm^3^ | 1.660 | 1.615 |
| µ, mm^–1^ | 0.745 | 0.975 |
| *F*(000) | 3272 | 1144 |
| θ Range, deg | 2.1-25.0 | 2.2-25.0 |
| Reflection Collected | 11402 | 4118 |
| Independent reflections (*R*_int_) | 0.067 | 0.072 |
| Reflections with *I* > 2σ(*I*) | 7877 | 3157 |
| Number of parameters | 955 | 358 |
| *R*_1_, *wR*_2_ (*I* > 2σ(*I*))^*^ | 0.0498, 0.1018 | 0.0428, 0.1023 |
| *R*_1_, *wR*_2_ (all data)^**^ | 0.0852, 0.1161 | 0.0601, 0.1092 |

* *R* = ∑(*F*_o_ – *F*_c_)/∑(F_o_), ** *wR*_2_ = {∑[*w*(*F*_o_^2^ – *F*_c_^2^)^2^]/∑(*F*_o_^2^)^2^}^1/2^.

**Table S3.** Selected bond distances (Å) and angles (deg) for **1-2**

| **1** | | | | | |
| --- | --- | --- | --- | --- | --- |
| Cd(1)-O(1) | 2.118(4) | Cd(1)-O(13) | | | 2.297(3) |
| Cd(1)-O(14) | 2.350(3) | Cd(1)-N(1) | | | 2.281(4) |
| Cd(1)-N(2) | 2.327(4) | Cd(2)-O(3) | | | 2.280(3) |
| Cd(2)-O(4) | 2.392(3) | Cd(2)-N(1) | | | 2.312(4) |
| Cd(2)-N(4) | 2.289(4) |  | | |  |
| **2** | | | | | |
| Cd(1)-O(1) | 2.403(3) | Cd(1)-O(2) | | | 2.344(3) |
| Cd(1)-N(1) | 2.374(5) | Cd(1)-N(2) | | | 2.338(4) |
| Cd(1)-O(4)#1 | 2.304(4) | Cd(1)-O(3)#2 | | | 2.321(3) |
|  | | | | | |
| **1** | | | | | |
| O(1)-Cd(1)-O(13) | 104.13(13) | | O(1)-Cd(1)-O(14) | 133.40(13) | |
| O(1)-Cd(1)-N(1) | 142.54(15) | | O(1)-Cd(1)-N(2) | 113.03(14) | |
| O(13)-Cd(1)-O(14) | 56.60(12) | | O(13)-Cd(1)-N(1) | 88.89(13) | |
| O(13)-Cd(1)-N(2) | 139.04(13) | | O(14)-Cd(1)-N(1) | 83.26(14) | |
| O(14)-Cd(1)-N(2) | 85.14(13) | | N(1)-Cd(1)-N(2) | 71.03(14) | |
| O(3)-Cd(2)-O(4) | 55.94(12) | | O(3)-Cd(2)-N(3) | 137.83(13) | |
| O(3)-Cd(2)-N(4) | 87.01(13) | | O(4)-Cd(2)-N(3) | 85.35(13) | |
| O(4)-Cd(2)-N(4) | 83.93(13) | | N(3)-Cd(2)-N(4) | 71.43(14) | |
| **2** | | | | | |
| O(1)-Cd(1)-O(2) | 54.98(11) | | O(1)-Cd(1)-N(1) | 101.94(15) | |
| O(1)-Cd(1)-N(2) | 85.22(13) | | O(1)-Cd(1)-O(4)#1 | 132.65(12) | |
| O(1)-Cd(1)-O(3)#2 | 90.62(12) | | O(2)-Cd(1)-N(1) | 110.72(15) | |
| O(2)-Cd(1)-N(2) | 140.00(13) | | O(2)-Cd(1)-O(4)#1 | 78.03(12) | |
| O(2)-Cd(1)-O(3)#2 | 98.73(12) | | N(1)-Cd(1)-N(2) | 70.35(16) | |
| O(4)#1-Cd(1)-N(1) | 88.37(17) | | O(3)#2-Cd(1)-N(1) | 150.12(14) | |
| O(4)#1-Cd(1)-N(2) | 140.59(14) | | O(3)#2-Cd(1)-N(2) | 84.04(13) | |

Symmetry Cddes: **For 2**: #1 = -1+x, y, z; #2 = 2-x, y, 1/2-z.
